# Novel Fas-TNFR chimeras that prevent Fas ligand-mediated kill and signal synergistically to enhance CAR T cell efficacy

**DOI:** 10.1016/j.omtn.2023.04.017

**Published:** 2023-04-25

**Authors:** Callum McKenzie, Mohamed El-Kholy, Farhaan Parekh, Mathew Robson, Katarina Lamb, Christopher Allen, James Sillibourne, Shaun Cordoba, Simon Thomas, Martin Pule

**Affiliations:** 1 Autolus Therapeutics, London W12 7FP, UK; 2 Department of Haematology, UCL Cancer Institute, University College, 72 Huntley Street, London WC1E 6DD, UK

**Keywords:** MT: Oligonucleotides: Therapies and Applications, apoptosis, Fas ligand, adoptive cell therapy, CAR T cells, synthetic biology, immunotherapy, T cell signaling, cancer therapy

## Abstract

The hostile tumor microenvironment limits the efficacy of adoptive cell therapies. Activation of the Fas death receptor initiates apoptosis and disrupting these receptors could be key to increasing CAR T cell efficacy. We screened a library of Fas-TNFR proteins identifying several novel chimeras that not only prevented Fas ligand-mediated kill, but also enhanced CAR T cell efficacy by signaling synergistically with the CAR. Upon binding Fas ligand, Fas-CD40 activated the NF-κB pathway, inducing greatest proliferation and IFN-γ release out of all Fas-TNFRs tested. Fas-CD40 induced profound transcriptional modifications, particularly genes relating to the cell cycle, metabolism, and chemokine signaling. Co-expression of Fas-CD40 with either 4-1BB- or CD28-containing CARs increased *in vitro* efficacy by augmenting CAR T cell proliferation and cancer target cytotoxicity, and enhanced tumor killing and overall mouse survival *in vivo*. Functional activity of the Fas-TNFRs were dependent on the co-stimulatory domain within the CAR, highlighting crosstalk between signaling pathways. Furthermore, we show that a major source for Fas-TNFR activation derives from CAR T cells themselves via activation-induced Fas ligand upregulation, highlighting a universal role of Fas-TNFRs in augmenting CAR T cell responses. We have identified Fas-CD40 as the optimal chimera for overcoming Fas ligand-mediated kill and enhancing CAR T cell efficacy.

## Graphical abstract

**Figure:**
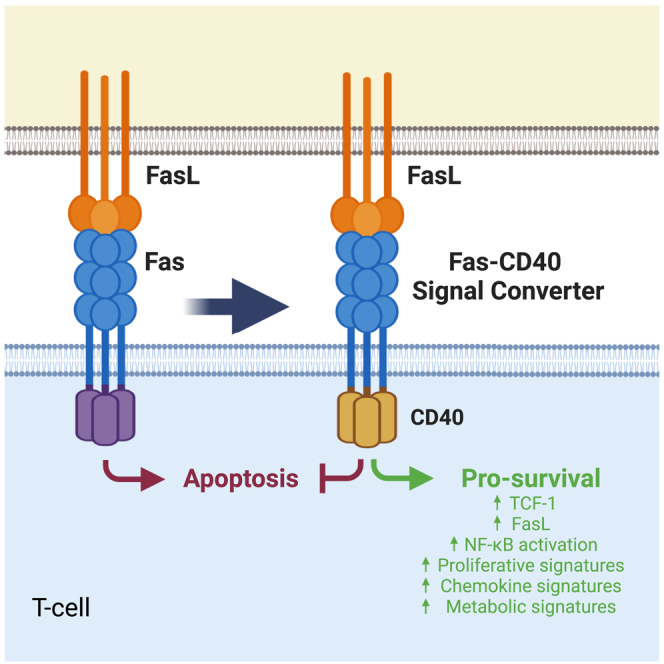

## Teaser

Pule and colleagues screened a library of Fas-TNFR chimeras in human T cells identifying Fas-CD40 as the optimal chimera for converting the FasL-inducing death response into a proliferative signal, in addition to enhancing CAR T cell cytotoxicity.

## Introduction

Adoptive transfer of chimeric antigen receptor (CAR) T cells has seen remarkable success in the treatment of relapsed/refractory hematological cancers; however, approximately 60% of patients eventually relapse, partly due to the hostile tumor microenvironment (TME).^1^ Extending these clinical successes to solid tumor indications is more challenging due to an even more complex and immunosuppressive TME.^1^^,^^2^^,^^3^

**Figure 1:**
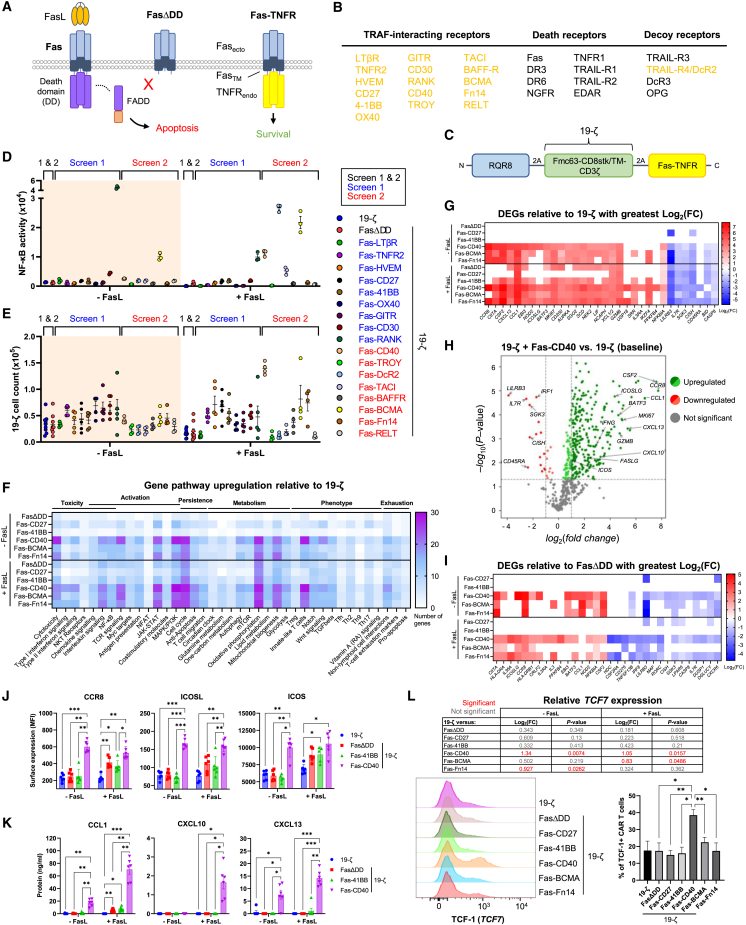
Fas-CD40 activates NF-κB and induces strong proliferation upon FasL binding (A) Left: upon binding FasL, Fas trimerization recruits FADD, initiating apoptosis. Middle: FasΔDD acts as a decoy receptor to FasL by being unable to recruit FADD. Right: schematic of Fas-TNFR structure; the ectodomain and transmembrane domain of Fas are fused to the endodomains of TNFRs. Upon FasL binding the Fas-TNFR chimera converts the death signal into a survival/growth signal. (B) Members of the TNFR superfamily. Those highlighted in gold were included in the Fas-TNFR screen. (C) Schematic of polycistronic transgene transduced into human T cells. 19-ζ: Fmc63 binder fused to the endodomain of CD3ζ via a CD8 stalk/transmembrane domain. (D) NF-κB reporter Jurkat cells transduced to express either 19-ζ alone or co-express FasΔDD or the Fas-TNFRs were cultured with or without immobilized recombinant FasL (20 μg/mL) overnight and NF-κB activity was measured. Experiment performed with technical triplicates, error bars are SEM. (E) Human T cells (5 × 10^4^) expressing 19-ζ and FasΔDD or the Fas-TNFRs were cultured with or without immobilized recombinant FasL (20 μg/mL) for 5 days, at which point cell counts were analyzed by flow cytometry. Due to the large list of Fas-TNFR chimeras, they were tested over two separate experiments (screens 1 and 2) with the data being compiled onto one graph. The conditions were identical between screens having the same 19-ζ and FasΔDD controls. Five independent donors were tested in screen 1 and four independent donors were tested in screen 2, error bars are SEM. (F) Human T cells from five independent donors were transduced to express 19-ζ or co-express FasΔDD or the stated Fas-TNFRs and then cultured with or without immobilized recombinant FasL (20 μg/mL) for 3 days, at which point RNA was extracted and analyzed using the nCounter NanoString platform with the CAR-T Characterization Panel. 19-ζ cells co-expressing FasΔDD or the Fas-TNFRs were normalized to 19-ζ alone, and the number of significantly (p < 0.05) upregulated differentially expressed genes (DEGs) were categorized by pathway involvement. (G) Significant DEGs relative to 19-ζ with greatest Log_2_ fold change (FC) from the experiment described in (F). (H) Volcano plot from the experiment described in (F) of Fas-CD40-19-ζ cells compared with 19-ζ alone after incubation with immobilized FasL. (I) Significant DEGs relative to FasΔDD-19-ζ with greatest Log_2_(FC) from the experiment described in (F). (J and K) 19-ζ cells were cultured in the presence or absence of immobilized FasL (20 μg/mL) for 5 days and then stained for CCR8, ICOSL, and ICOS expression by flow cytometry (I), or the cell culture supernatant analyzed for CCL1, CXCL10, and CXCL13 secretion (J). Six independent donors tested, error bars are SEM, ∗p < 0.05, ∗∗p < 0.01, ∗∗∗p < 0.001, two-way ANOVA. (L) Top: *TCF7* expression from the experiment described in (F). Bottom left: 19-ζ cells were stained for TCF-1, representative flow cytometry plots from one donor. Bottom right: TCF-1 expression from three independent donors, error bars are SEM, ∗p < 0.05, ∗∗p < 0.01, two-way ANOVA.

The Fas/Fas ligand (FasL) pathway is a key inhibitory checkpoint contributing to the immunosuppressive TME.^4^^,^^5^^,^^6^^,^^7^ Fas is a member of the tumor necrosis factor receptor (TNFR) superfamily and comprises one of eight TNFR death receptors.^8^ Upon binding FasL, Fas trimerizes allowing for binding of the adaptor protein, Fas-associated death domain (FADD), to the intracellular death domains of Fas via homotypic interactions.^9^ Pro-caspase-8 then binds FADD via death effector domains, creating the death-inducing signaling complex, and is then cleaved to activate downstream executioner caspases, initiating apoptosis (Figure 1A).

T cells constitutively express Fas and are consequently vulnerable to FasL-mediated apoptosis. The *FASLG* gene and FasL protein are overexpressed in many cancers, either by cancer cells themselves or by cells constituting the TME, such as regulatory T cells (Tregs), myeloid-derived suppressor cells (MDSCs), cancer-associated fibroblasts (CAFs), and tumor endothelial cells.^5^^,^^6^ Moreover, T cells upregulate FasL upon activation, inducing fratricide, an effect particularly observed with third-generation CARs.^10^^,^^11^ Therefore, the Fas/FasL checkpoint can limit the efficacy of adoptive T cell therapy.

Several strategies to overcome FasL in immunotherapy have been explored. Therapeutic monoclonal antibodies that block Fas or FasL effectively prevent FasL-mediated T cell loss; however, FasL-mediated killing of tumor is concomitantly compromised.^12^^,^^13^^,^^14^ Adoptive immunotherapy with engineered immune cells affords more discrete methods: disruption of Fas expression by small interfering RNAs or CRISPR-Cas9 is effective.^15^^,^^16^ An alternative strategy is expression of non-functional Fas, which competes with native Fas. This latter strategy includes a truncated Fas receptor lacking the death domain (FasΔDD) or a chimeric Fas-41BB protein.^5^^,^^17^^,^^18^^,^^19^ Expression of FasΔDD or Fas-41BB rescues FasL-mediated apoptosis. The Fas-41BB chimera additionally converts the death signal into a pro-survival 4-1BB signal by activating NF-κB and mitogen-activated protein kinase (MAPK) pathways via TNFR-associated factors (TRAFs).^20^

There are many other members of the TNFR superfamily apart from 4-1BB that provide co-stimulatory signals that, due to differential TRAF activation, may be qualitatively different. In this paper we perform a functional assessment of Fas-TNFR chimeric proteins in the context of human T cells. We identify several novel Fas-TNFR chimeras that co-stimulate CAR T cells, delivering enhanced target cytotoxicity and CAR T cell persistence/proliferation compared with FasΔDD and the Fas-41BB chimera. In particular, Fas-CD40 optimally enhanced CAR T cell efficacy when co-expressed with either 4-1BB- or CD28-containing CARs. Moreover, we demonstrate that a major source of FasL for Fas-TNFR activation derives from T cells themselves, highlighting a universal role of Fas-TNFRs to augment CAR T cell therapy.

## Results

### Screening of Fas-TNFRs reveals Fas-CD40 as a potent inducer of proliferation upon binding FasL

We first created a set of Fas-TNFR chimeras comprising the ectodomain and transmembrane domain of Fas fused to the endodomains of pro-survival TRAF-interacting TNFRs, as well as the endodomain of the TRAIL decoy receptor, TRAIL-R4/DcR2^21^ (Figures 1A and 1B). The Fas-TNFR chimeras (highlighted in gold in Figure 1B) were co-expressed in primary human T cells with the RQR8 suicide/sort marker^22^ and a first-generation CD19-targeting CAR (19-ζ; Figure 1C), and were screened for their ability to resist FasL-mediated cell death and to alter T cell activity upon binding FasL. Screening was performed in two separate experiments due to the large numbers of Fas-TNFR chimeras.

Upon binding FasL, Fas-CD40, Fas-DcR2, Fas-TACI, and Fas-BCMA induced NF-κB activity, where Fas-DcR2 surprisingly displayed greatest induction (Figures 1D and S1A). Fas-BCMA, Fas-CD30, and, to a lesser extent, Fas-CD40, exhibited constitutive NF-κB activity (Figure 1D). Fas-RANK resulted in high constitutive NF-κB activity, which paradoxically decreased in the presence of FasL (Figures 1D and S1A). Fas-CD40 induced the greatest proliferation upon binding FasL with a relative fold difference of 3.4 (Figures 1E and S1B). Fas-BCMA, Fas-HVEM, Fas-CD27, Fas-Fn14, Fas-41BB, and Fas-BAFFR also induced FasL-dependent proliferation. Corresponding increases of IFN-γ secretion were observed for these Fas-TNFRs (Figure S1C). Fas-LTβR and Fas-CD30 expression resulted in constitutive IFN-γ secretion (Figure S1C), despite not resulting in basal or induced proliferation (Figures 1E and S1B). No IL-2 secretion was induced by any of the Fas-TNFRs in response to FasL (Figure S1D). Fas-TNFR chimeras that induced proliferation upon binding FasL (Fas-HVEM, Fas-CD27, Fas-41BB, Fas-CD40, Fas-BAFFR, Fas-BCMA, and Fas-Fn14; Figure S1B) were selected for further study and assayed in response to FasL using six different donors, which confirmed the earlier findings (Figure S1E). We continued our investigations with the following chimeras: Fas-CD27, Fas-41BB, Fas-CD40, Fas-BCMA, and Fas-Fn14.

### Fas-CD40 induces profound transcriptional changes

We next investigated how the Fas-TNFRs affected gene transcription using the nCounter NanoString platform. Comparative analyses identifying differentially expressed genes (DEGs) of transcripts relative to 19-ζ-expressing cells revealed two clusters: (1) FasΔDD, Fas-CD27, and Fas-41BB and (2) Fas-CD40, Fas-BCMA, and Fas-Fn14 (Figures 1F, 1G, and S2A; Table S1). Under basal conditions, cluster 2 induced greater gene transcription compared with cluster 1, and in the presence of FasL transcriptional increases were observed across both clusters, consistent with markers of functional activation observed above (Figures 1D, 1E, and S1C). Upregulated DEGs specifically identified in cluster 2 related to: (1) T cell cytotoxicity, (2) cell cycle, (3) chemokine and interleukin signaling, (4) JAK-STAT, MAPKs, phosphoinositide 3-kinase (PI3K), and NF-κB pathways, (5) co-stimulatory molecules, (6) T cell memory, and (7) oxidative phosphorylation, mitochondrial biogenesis, lipid metabolism, and glycolysis (Figures 1F and 1G; Table 1).

**Table 1:** Upregulated DEGs specific to Fas-CD40, Fas-Fn14, and Fas-BCMA expression

Gene pathway	Genes
T cell cytotoxicity	*CSF2*, *IFNG*, *GZMB*, *PRF1*, *FASLG*
JAK-STAT signaling	*LIF*, *STAT1*, *STAT3*, *STAT5A*, *SOCS4*, *CRLF2*
MAPK/PI3K signaling	*CDC42*, *PIK3R1*, *PIK3R2*, *PIK3R3*, *RAC2*, *MAPK3*, *MAP2K2*, *MAP3K14*
Cell cycle	*NSD2*, *AURKA*, *SGO2*, *NEK2*, *MKI67*, *BUB1*, *NCAPH*
Oxidative phosphorylation	*NDUFA1*, *NDUFA2*, *ATP5MF*, *IDH3A*, *COX4I1*, *COX5B*, *COX6B1*, *COX6C*, *COX7A2*, *COX7B*, *COX7C*
Mitochondrial biogenesis	*SLC25A6*, *HSPE1*, *NAA20*, *COX19*
Lipid metabolism	*HMGCR*, *SCD*, *MID1IP1*, *ACACA*, *ACSF2*
Glycolysis	*PFKFB4*, *LDHA*, *PGAM1*, *PGK1*, *PKM*
T cell memory	*CD45RO*, *SELL*, *LEF1*, *BATF3*, *TCF7*
Interleukin signaling	*EBI3*, *IL2RA*, *IL2RB*, *IL2RG*, *IL3*, *IL3RA*, *IL12RB2*, *IL21R*, *IL32*, *IL36A*
Chemokine signaling	*CCR8*, *CXCR3*, *CXCR4*, *CCL1*, *CXCL10*, *CXCL13*, *XCL1/2*, *CCR7*
NF-κB pathway	*NFKBIA*, *NFKB2*, *BCL2*, *BCL2L1*, *UBE2I*, *PARP1*, *IKBKE*, *RELA*
Co-stimulatory molecules	*TNFRSF4*, *TNFRSF9*, *TNFRSF18*, *ICOSLG*, *ICOS*, *CD80*, *CD27*

Selected list of upregulated significant (p < 0.05) DEGs relating to their pathway involvement. T cells were treated as described in Figure 1F.

Within cluster 2, Fas-CD40 displayed greater differential gene transcription, which is exemplified by the number of uniquely transcribed genes (Figure 1H; Table 2). Notably, Fas-CD40 induced greater gene transcription for chemokine receptors: *CCR8*, *CXCR3*, and *CXCR4*; chemokine ligands: *CCL1* (encodes ligand for CCR8), *CXCL10* (encodes ligand for CXCR3), and *CXCL13*; and *ICOSLG* (encodes ligand for ICOS) (Figure 1H). Downregulated DEGs included inhibitory checkpoints (*CISH* and *LILRB3*) and pro-apoptotic markers (*BID* and *CASP8*) (Figures 1G and 1H). Fas-TNFR chimera surface expression did not correlate with transcriptional clustering, excluding this as a cause for transcriptional differences between chimeras (Figure S2C).

**Table 2:** List of uniquely transcribed genes relative to 19-ζ expression

	-FasL						+FasL					
	Upregulated			Downregulated			Upregulated			Downregulated		
	Gene	Log_2_ FC	p value	Gene	Log_2_ FC	p value	Gene	Log_2_ FC	p value	Gene	Log_2_ FC	p value
FasΔDD	MX1-mRNA	1.29	0.0384	none			TNF-mRNA	1.24	0.00718	none		
							TICAM1-mRNA	1.1	0.0262			
							FYN-mRNA	0.292	0.04			
							CTNNA1-mRNA	1.67	0.0456			
Fas-CD27	JAK2-mRNA	1.04	0.00251	CD8A-mRNA	−1.26	0.000314	none			TYROBP-mRNA	−3.05	0.0332
	JAK1-mRNA	0.85	0.00252	NFIL3-mRNA	−1.24	0.00787				KLRB1-mRNA	−1.21	0.0344
	RORA-mRNA	0.931	0.00555	NDUFB9-mRNA	−1.29	0.0115				TOLLIP-mRNA	−0.516	0.0396
	XAF1-mRNA	0.943	0.00662	MAP2K2-mRNA	−1.29	0.012						
	STAT5B-mRNA	0.766	0.0176	MIF-mRNA	−1.4	0.0156						
	CCL5-mRNA	0.765	0.0313	SH2D1A-mRNA	−0.825	0.0236						
	SP100-mRNA	0.332	0.0348									
	TGFBR2-mRNA	0.535	0.0366									
	TRIM22-mRNA	0.653	0.0443									
	VAV1-mRNA	0.783	0.0453									
Fas-41BB	SMAD3-mRNA	0.601	0.016	none			CD160-mRNA	3.56	0.00149	AFDN-mRNA	−1.02	0.0245
	PTPRC-mRNA	0.286	0.0427							VSIR-mRNA	−0.81	0.0297
	KLRK1-mRNA	0.456	0.0486							MAML2-mRNA	−0.485	0.047
										DVL2-mRNA	−0.431	0.0473
Fas-CD40	CCNC-mRNA	1.07	4.36E-05	PDK1-mRNA	−0.643	0.041	IL3RA-mRNA	4.04	0.000788	RORC-mRNA	−1.04	0.0197
	ICOSLG-mRNA	4.67	4.99E-05	RORC-mRNA	−0.84	0.0423	PFKFB4-mRNA	4.82	0.00245			
	ATP5PD-mRNA	0.71	0.000794				CXCL10-mRNA	3.26	0.00572			
	SERINC3-mRNA	0.767	0.00163				OASL-mRNA	2.98	0.0123			
	CRLF2-mRNA	3.22	0.00164				AKT2-mRNA	0.518	0.0127			
	SDHB-mRNA	0.656	0.00265				RPTOR-mRNA	1.23	0.0152			
	ALDOA-mRNA	1.17	0.00292				TNFRSF4-mRNA	1.1	0.0177			
	PYCR2-mRNA	0.88	0.00314				CTNND1-mRNA	1.62	0.0177			
	PECAM1-mRNA	2.48	0.00318				SERINC3-mRNA	0.428	0.0199			
	IL12RB2-mRNA	1.19	0.0036				PPAT-mRNA	1.36	0.0248			
	NFIL3-mRNA	1.11	0.00364				STAT1-mRNA	1.27	0.0273			
	MAP3K14-mRNA	0.844	0.00399				PFKP-mRNA	0.565	0.0302			
	CD3E-mRNA	0.812	0.00443				ATP6V1F-mRNA	0.724	0.0305			
	UBA5-mRNA	1.4	0.00461				HLA-E-mRNA	0.603	0.0336			
	SLC2A1-mRNA	2.15	0.00494				ITGB2-mRNA	1.13	0.0354			
	MIF-mRNA	1.2	0.00553				FCGR3A/B-mRNA	2.31	0.0367			
	TNFSF9-mRNA	2.06	0.00573				PRDM1-mRNA	1.58	0.0384			
	IL4R-mRNA	1.59	0.00622				IFI6-mRNA	2.69	0.0386			
	CD80-mRNA	1.86	0.00712				TRIM33-mRNA	0.367	0.0422			
	MTHFS-mRNA	0.837	0.0072				GATA3-mRNA	0.81	0.0431			
	ADAR-mRNA	1.03	0.00735				IKBKE-mRNA	1.02	0.0431			
	HDAC7-mRNA	1	0.00788				PPP2R5D-mRNA	2.76	0.0485			
	RELA-mRNA	0.813	0.00861									
	LCK-mRNA	0.612	0.00871									
	COX5B-mRNA	1.05	0.00983									
	GATA3-mRNA	1.25	0.0104									
	MAP3K7-mRNA	0.605	0.0105									
	RAC2-mRNA	1.16	0.0152									
	GRPEL1-mRNA	1.09	0.016									
	OAS3-mRNA	1.74	0.0172									
	ACADVL-mRNA	0.634	0.0176									
	PPP2R5D-mRNA	1.63	0.0196									
	TNFRSF4-mRNA	1.19	0.0206									
	IRF5-mRNA	2.31	0.0228									
	ITGB2-mRNA	1.35	0.023									
	COX7C-mRNA	0.749	0.0237									
	CD45R0-mRNA	1.3	0.0237									
	MDH2-mRNA	1.12	0.0242									
	CD7-mRNA	0.786	0.0248									
	NFAT5-mRNA	1.55	0.03									
	TRIM33-mRNA	0.509	0.0317									
	DHRS4-mRNA	0.567	0.0322									
	ACOT2-mRNA	0.99	0.034									
	PRICKLE3-mRNA	0.788	0.0343									
	TET2-mRNA	0.923	0.037									
	MAP2K2-mRNA	0.762	0.0422									
	SEC22B-mRNA	0.473	0.0435									
	SH3BP2-mRNA	1.05	0.0442									
	TOLLIP-mRNA	0.517	0.0449									
	STK11-mRNA	0.668	0.0456									
	TNFRSF10B-mRNA	0.914	0.0466									
	MAPK3-mRNA	0.734	0.0481									
	PSMB10-mRNA	0.722	0.0485									
	AKT1-mRNA	0.552	0.0486									
	MR1-mRNA	1.22	0.0499									
Fas-BCMA	SRR-mRNA	2.88	0.00012	SERINC1-mRNA	−0.372	0.0162	LAT-mRNA	0.993	0.00325	none		
	GFER-mRNA	2.27	0.00793	ADORA2A-mRNA	−1.5	0.0162	SH3BP2-mRNA	0.894	0.0346			
	TNF-mRNA	1.07	0.0245	IRF9-mRNA	−0.374	0.0406	BATF-mRNA	0.942	0.0369			
	CCL4/L1-mRNA	1.37	0.0414	ATG7-mRNA	−0.438	0.046						
Fas-Fn14	CX3CR1-mRNA	2.12	0.00385	CALM1-mRNA	−0.499	0.0458	PYCR3-mRNA	2.85	1.77E-07	RORA-mRNA	−0.638	0.0149
	SOCS5-mRNA	1.08	0.0254				IL3-mRNA	2.59	0.0273	CCR2-mRNA	−2.25	0.0169
										CCR6-mRNA	−1.52	0.0207
										EGR1-mRNA	−1.1	0.0455

T cells were treated as described in Figure 1F. TCR diversity genes included in the nCounter CAR-T Characterization Panel have been removed from this list. FC, fold change.

Comparative analysis of Fas-TNFRs versus FasΔDD revealed similar upregulated DEGs compared with 19-ζ such as those related to NF-κB, interleukin, and chemokine signatures (Figures 1I and S2D; Table S2). However, upon binding FasL, there were greater downregulated DEGs in cluster 2, which included markers of T cell inhibition (*CD200*, *CTLA4*, *LILRB3*, *CD276*, and *CD84*), senescence (*KLRG1*), exhaustion (*TOX*), and apoptosis (*GADD45B*, *CASP8*, and *BID*).

We confirmed increased protein expression of CCR8, ICOSL, and ICOS upon Fas-CD40 expression (Figure 1J), in addition to increased secretion of CCL1, CXCL10, and CXCL13 (Figure 1K), correlating with our transcriptomic analysis. Interestingly, Fas-CD40 was unique in upregulating TCF-1, as well as its corresponding gene, *TCF7* (Figures 1L and S2E; Table 1), which is a key marker for T cell stemness, memory, survival, and proliferation.^23^ Also correlating with our transcriptomic data, we confirmed that Fas-CD40 activated the MAPKs (ERK and p38) and upregulated protein expression of PI3K and STAT-1, -3, and -5 (Figure S3). Fas-BCMA and Fas-Fn14 also activated ERK, albeit to a lesser extent, with Fas-Fn14 additionally upregulating STAT-1 and -3 (Figure S3).

### Fas-TNFR chimeras protect from FasL-mediated kill

**Figure 2:**
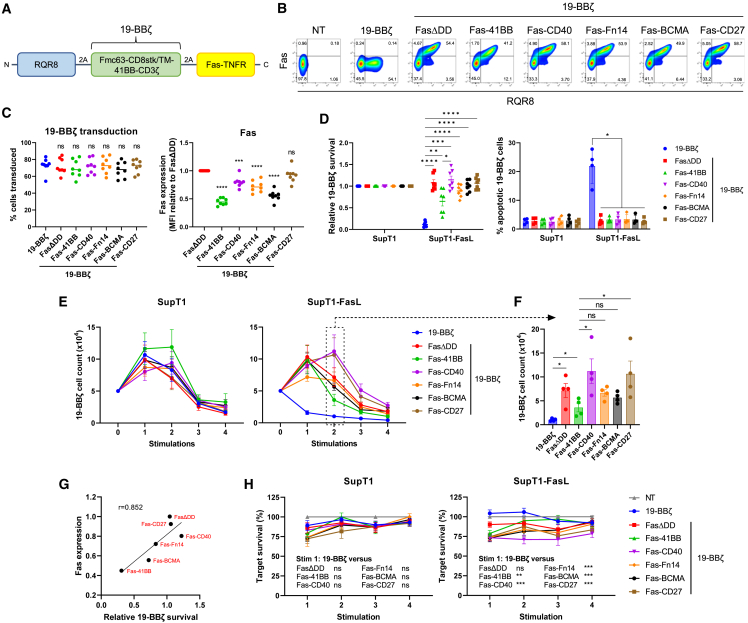
Fas-TNFRs rescue FasL-mediated kill (A) Schematic of polycistronic transgene transduced into human T cells. 19-BBζ:Fmc63 binder fused to the endodomains of 4-1BB and CD3ζ via a CD8 stalk/transmembrane domain. (B) Representative flow cytometry plots from one human T cell donor transduced to express either 19-BBζ alone or co-express FasΔDD or the stated Fas-TNFRs. Statistical analyses from multiple donors are shown in (C). (C) Left: transduction percentages of T cells from eight independent donors; ns, non-significant, one-way ANOVA (Dunnett’s multiple comparisons test relative to 19-BBζ). Right: median fluorescence intensity (MFI) of the Fas-TNFRs relative to FasΔDD MFI, measured from the top right quadrant in (B). Eight independent donors tested, mean being shown, ∗∗∗p < 0.001, ∗∗∗∗p < 0.0001; ns, non-significant, one-way ANOVA (Dunnett’s multiple comparisons test relative to FasΔDD). (D) 19-BBζ cells were cultured with SupT1^FasKO^ or SupT1^FasKO^-FasL cells either for 72 h (left) or 5 h (right) at a 1:1 effector to target (E:T) ratio, at which point 19-BBζ cell survival or percentage of apoptotic cells (Annexin V^+^ 7AAD^−^) were calculated, respectively. Seven and four independent donors were tested for the cell survival and apoptotic analysis, respectively. Error bars are SEM, ∗p < 0.05, ∗∗p < 0.01, ∗∗∗p < 0.001, ∗∗∗∗p < 0.0001, two-way ANOVA. (E) 19-BBζ cells from four independent donors were stimulated with 5 × 10^4^ SupT1^FasKO^ or SupT1^FasKO^-FasL cells at an initial 1:1 E:T up to four times with cell counts being analyzed after each stimulation. (F) Cell numbers from (E) after the second round of SupT1^FasKO^-FasL stimulation. ∗p < 0.05; ns, non-significant, two-way ANOVA. (G) Mean average of relative Fas expression (from C) versus mean average of relative 19-BBζ survival (from second stimulation readout in (E) and Figure S4B), r, Pearson correlation coefficient. (H) From experiment described in (E), the percentage of surviving targets analyzed after each target stimulation. Error bars are SEM, ∗∗p < 0.01, ∗∗∗p < 0.001; ns, non-significant, two-way ANOVA.

We next assessed how efficiently the Fas-TNFRs could rescue FasL-mediated kill. We co-expressed the Fas-TNFRs with RQR8 and a second-generation CD19-targeting CAR (19-BBζ; Figure 2A). FasΔDD, Fas-CD27, and Fas-CD40 had the highest protein expression, respectively, followed by Fas-Fn14 then Fas-BCMA with Fas-41BB having the lowest (Figures 2B and 2C). Upon co-culture with SupT1 cells engineered to express FasL (Figure S4A), Fas-41BB could only partially rescue cell death as measured by cell survival; however, the percentage of apoptotic cells was indistinguishable from SupT1 control cells (Figure 2D). FasΔDD and the other Fas-TNFRs fully rescued FasL-mediated cell death. Upon repeated SupT1-FasL challenge, Fas-CD40, Fas-CD27, and FasΔDD completely rescued FasL-mediated cell death, with Fas-CD40 and Fas-CD27 additionally inducing CAR T cell proliferation (Figures 2E, 2F, and S4B), consistent with the initial screen. In contrast, Fas-41BB, Fas-BCMA, and Fas-Fn14 only partially rescued cell survival. Fas-TNFR protein expression correlated with 19-BBζ survival (Figure 2G). Fas-TNFR-19-BBζ CAR T cells did not proliferate autonomously when co-cultured with CD19^−^ SupT1 cells (Figure 2E), nor did they increase tonic cytotoxicity relative to 19-BBζ alone (Figure 2H). The level of cytotoxicity slightly increased against SupT1-FasL cells (Figure 2H).

### Fas-CD40, Fas-BCMA, Fas-CD27, and Fas-Fn14 enhance 19-BBζ CAR efficacy

**Figure 3:**
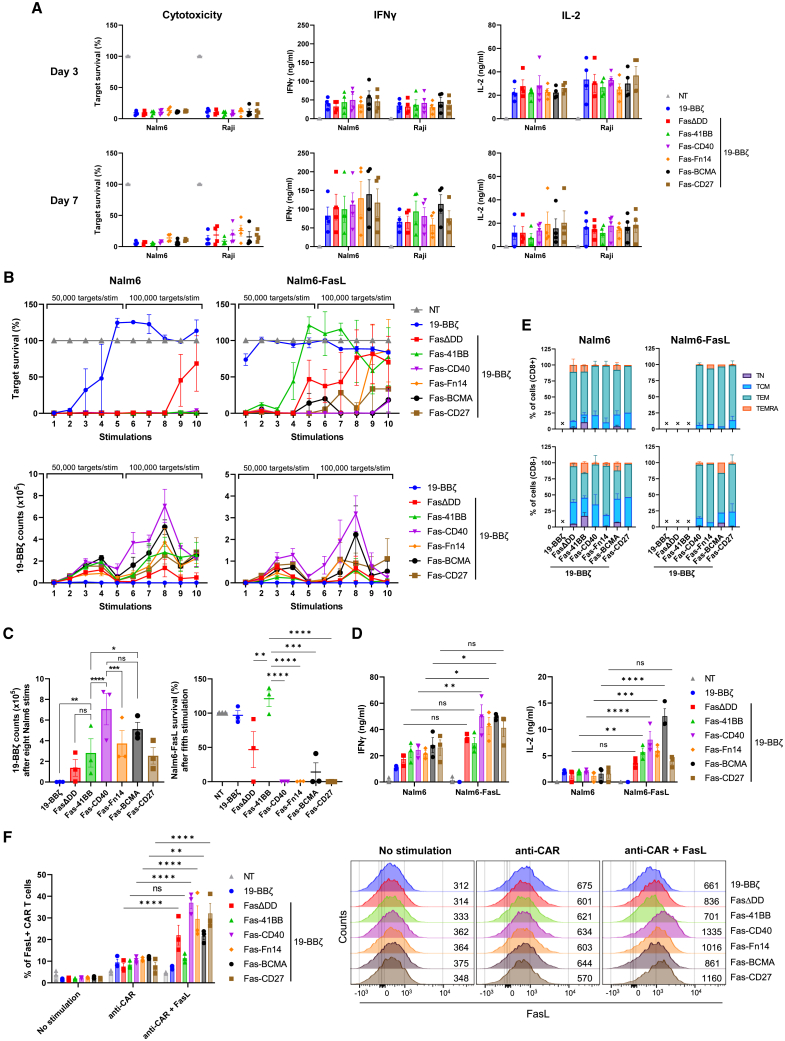
Fas-TNFRs augment 19-BBζ CAR efficacy (A) 19-BBζ cells co-expressing FasΔDD or the Fas-TNFRs were cultured with Nalm6 or Raji cells for 3 or 7 days at an E:T ratio of 1:4, measuring for target survival (left), secretion of IFN-γ (middle), and IL-2 (right). Four independent donors tested, error bars are SEM. (B) 19-BBζ cells from three independent donors were stimulated up to 10 times with either Nalm6^FasKO^ or Nalm6^FasKO^-FasL cells at a starting 1:8 E:T ratio, measuring for target survival and 19-BBζ cell counts after each stimulation. Effectors were stimulated with 50,000 targets for the first five stimulations and 100,000 targets for the final five stimulations, error bars are SEM. (C) Left: 19-BBζ cell counts after the eighth round of Nalm6^FasKO^ stimulation as described in (B). Right: relative target survival of Nalm6^FasKO^-FasL cells after the fifth round of stimulation as described in (B). ∗p < 0.05, ∗∗p < 0.01, ∗∗∗p < 0.001, ∗∗∗∗p < 0.0001; ns, non-significant, two-way ANOVA, error bars are SEM. (D) Cell culture supernatants after the first round of target stimulation from the experiment described in (B) were analyzed for IFN-γ and IL-2. ∗p < 0.05, ∗∗p < 0.01, ∗∗∗p < 0.001, ∗∗∗∗p < 0.0001; ns, non-significant, two-way ANOVA, error bars are SEM. (E) T cell memory phenotypes were analyzed for CD8 (top) and CD4 (bottom) cells after the seventh stimulation from the restimulation experiment described in (B). Error bars are SEM, an “X” denotes where too few cells were present to accurately determine memory phenotype. (F) Left: percentage of CAR T cells expressing FasL after being cultured for 48 h with either an immobilized anti-CD19 CAR idiotype antibody alone (anti-fmc63; 2 μg/mL) or also with immobilized FasL (2 μg/mL). Right: representative flow cytometry plots from one donor from the graph on the left with the MFI being shown. Three independent donors tested, ∗∗p < 0.01, ∗∗∗∗p < 0.0001; ns, non-significant, two-way ANOVA, error bars are SEM.

We then assessed how the Fas-TNFRs affected 19-BBζ-mediated target cell cytotoxicity by co-culturing with multiple cancer cell lines. Fas-TNFR-19-BBζ cells exhibited equivalent cytotoxicity and secretion of IFN-γ and IL-2 against CD19^+^ Nalm6 and Raji cancer cells compared with 19-BBζ alone (Figures 3A and S5A). In addition, Fas-TNFR-19-BBζ cells demonstrated equivalent cytotoxicity against SupT1 cells engineered to express the CAR cognate antigen CD19, with no background killing observed against CD19^−^ SupT1 cells (Figure S5B). Expression of FasΔDD or the Fas-TNFRs did not alter the kinetics of programmed cell death protein 1 (PD-1) expression upon co-culture with Nalm6 target cells (Figure S5C).

To stress test the Fas-TNFR chimeras we set up an *in vitro* restimulation cytotoxicity assay, where 19-BBζ cells were serially challenged with Nalm6 cells and Nalm6 cells engineered to express FasL (Figure S5D). Expression of FasΔDD and the Fas-TNFRs enhanced 19-BBζ-mediated Nalm6 cytotoxicity, killing targets for all 10 stimulations (Figure 3B), with Fas-CD40 inducing greatest proliferation, a 113-fold increase from the initial 1:8 effector to target (E:T) seeding ratio (Figures 3B and 3C). Fas-CD40, Fas-BCMA, Fas-CD27, and Fas-Fn14 enhanced serial cytotoxicity against Nalm6-FasL cells compared with FasΔDD, with Fas-41BB impairing serial cytotoxicity, which again correlated with CAR T cell numbers (Figures 3B and 3C). Fas-CD40, Fas-Fn14, and Fas-BCMA induced greater IFN-γ and IL-2 secretion after one round of Nalm6-FasL stimulation compared with Nalm6 cells without exogenous FasL, with Fas-41BB also secreting greater IL-2 (Figure 3D). Equivalent memory phenotypes of the 19-BBζ cells were observed for the Fas-TNFRs, adopting either a central memory (TCM) or effector memory (TEM) phenotype, with Fas-41BB also tending to induce a greater terminal effector (TEMRA) phenotype (Figures 3E and S5E).

Expression of FasΔDD or the Fas-TNFRs did not perturb upregulation of CAR T cell-derived FasL upon CAR activation, which could otherwise remove an effective cancer-killing mechanism (Figure 3F). Interestingly, upon dual CAR/FasL stimulation Fas-CD40 induced greatest FasL upregulation, correlating with our transcriptomic analysis with Fas-CD40 upregulating *FASLG* transcription (Figure 1H; Tables 1 and S1); potentially creating a feedforward activation loop.

### Fas-CD40 also enhances 19-28ζ CAR efficacy

**Figure 4:**
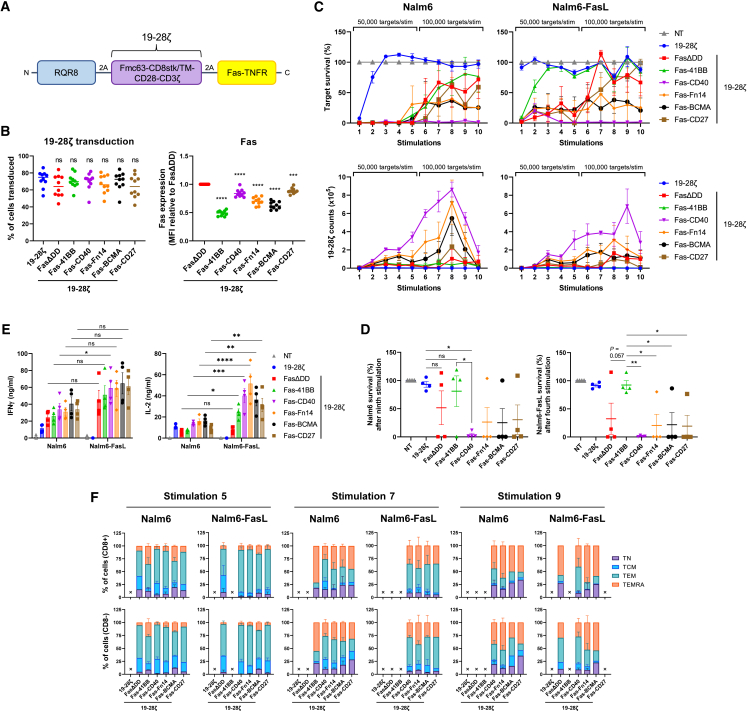
Fas-CD40 optimally enhances 19-28ζ CAR efficacy (A) Schematic of polycistronic transgene transduced into human T cells. 19-28ζ:Fmc63 binder fused to the endodomains of CD28 and CD3ζ via a CD8 stalk/transmembrane domain. (B) Left: transduction percentages of T cells from 10 independent donors; ns, non-significant, one-way ANOVA (Dunnett’s multiple comparisons test relative to 19-28ζ). Right: MFI of the Fas-TNFRs relative to FasΔDD MFI, measured from top right quadrant in Figure S6A. Ten independent donors tested, bars indicate means, ∗∗∗p < 0.001, ∗∗∗∗p < 0.0001, one-way ANOVA (Dunnett’s multiple comparisons test relative to FasΔDD). (C) 19-28ζ cells from four independent donors were stimulated up to 10 times with either Nalm6^FasKO^ or Nalm6^FasKO^-FasL cells at a starting 1:8 E:T ratio, measuring for target survival and 19-28ζ cell counts after each stimulation. Effectors were stimulated with 50,000 targets for the first five stimulations and 100,000 targets for the final five stimulations, error bars are SEM. (D) Relative target survival of Nalm6^FasKO^ (left) and Nalm6^FasKO^-FasL (right) cells after the ninth or fourth rounds of stimulation, respectively, as described in (C). ∗p < 0.05, ∗∗p < 0.01; ns, non-significant, two-way ANOVA, error bars are SEM. (E) Cell culture supernatants after the first round of target stimulation from the experiment described in (C) were analyzed for IFN-γ and IL-2. ∗p < 0.05, ∗∗p < 0.01, ∗∗∗p < 0.001, ∗∗∗∗p < 0.0001; ns, non-significant, two-way ANOVA, error bars are SEM. (F) T cell memory phenotypes were analyzed for CD8 (top) and CD4 (bottom) cells after the fifth, seventh, and ninth stimulations from the restimulation experiment described in (C). Error bars are SEM, an “X” denotes where too few cells were present to accurately determine memory phenotype.

We next investigated whether the co-stimulatory activity of the Fas-TNFRs would be affected if we replaced the co-stimulatory endodomain within the CAR from 4-1BB to CD28 (19-28ζ; Figure 4A). 19-28ζ was co-expressed in human T cells with RQR8 and the Fas-TNFRs (Figure S6A). As seen with 19-BBζ co-expression, FasΔDD displayed highest protein expression followed by Fas-CD27 and Fas-CD40, with Fas-41BB having the lowest expression (Figure 4B), which again correlated with the ability to rescue FasL-mediated cell death (Figures S6B and S6C). Fas-CD40, Fas-Fn14, and Fas-BCMA induced a slightly higher level of basal proliferation upon CD19^−^ SupT1 stimulation (Figure S6B), which correlated with increased tonic cytotoxicity; however, this was not sustained (Figure S6D). This is different to what was seen with 19-BBζ implying a difference in signal transduction. 19-28ζ cells co-expressing Fas-CD40, Fas-Fn14, and Fas-BCMA displayed greater cytotoxicity against SupT1-FasL cells, which was sustained with Fas-CD40 (Figure S6D).

Fas-CD40-19-28ζ cells exhibited greatest Nalm6 and Nalm6-FasL serial cytotoxicity, completely killing targets for all 10 stimulations (Figures 4C and 4D), which correlated with the level of CAR T cell proliferation (a 138-fold increase from the initial 1:8 E:T seeding ratio) and IFN-γ and IL-2 secretion (Figure 4E). The proliferative capacity of Fas-CD40-19-28ζ cells was not limitless, however, as CAR T cell proliferation decreased after the last two stimulations, as did the other Fas-TNFRs. Fas-Fn14, Fas-BCMA, and Fas-CD27 trended to augment target cytotoxicity similar to Fas-CD40; however, an outlier precluded definitive statistical analyses. Throughout the stimulations, Fas-CD40-19-28ζ cells maintained an earlier memory profile compared with the other Fas-TNFRs by having fewer CD45RA^+^CD62L^−^ TEMRA cells across both CD8 and CD4 populations (Figure 4F).

### Fas-CD40 optimally enhances GD2-28ζ CAR efficacy

We next investigated the functionality of the Fas-TNFRs in the context of a CAR targeting a different antigen, the disialoganglioside GD2, to confirm applicability across multiple CAR architectures. Fas-TNFRs (located at the N terminus) were co-expressed with RQR8 and a GD2-targeting CAR (GD2-28ζ; Figures S7A and S7B). Fas-CD27, Fas-CD40, and FasΔDD had highest protein expression (Figure S7C), with all Fas-TNFRs rescuing FasL-mediated apoptosis (Figure S7D). Without target stimulation, all Fas-TNFR-GD2-28ζ cells had equivalent memory phenotypes and exhaustion-associated marker expression (Figure S7E), and exhibited equivalent cytotoxicity against SupT1 cells engineered to express GD2 (Figure S7F). Upon serial target stimulation, Fas-CD40 optimally enhanced GD2-28ζ-mediated cytotoxicity against SupT1-GD2 and SupT1-GD2-FasL cells, which correlated with the level of CAR T cell proliferation (Figures S7G and S7H), similar to that observed with 19-28ζ; however, with the effects less pronounced. Upon serial target stimulation, Fas-CD40-GD2-28ζ cells trended to have an earlier memory phenotype compared with GD2-28ζ or the other Fas-TNFRs, as seen by fewer TEMRA and greater effector memory cells (Figure S7I), and CD4^+^ Fas-CD40-GD2-28ζ cells expressed fewer exhaustion-associated markers (Figure S7J).

### CAR T cell-derived FasL may be an additional source for Fas-TNFR activation

**Figure 5:**
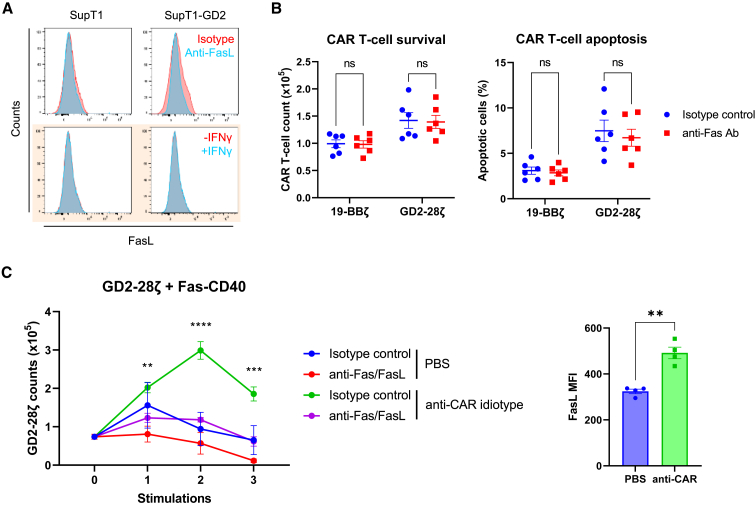
Fas-CD40 enhances CAR T cell proliferation without exogenous source of FasL (A) Top: SupT1 and SupT1-GD2 (Fas^+/+^) cells were surface stained with an anti-FasL antibody or an isotype control antibody. Bottom (in colored box): SupT1 or SupT1-GD2 cells were treated with vehicle (PBS) or IFN-γ (100 ng/mL) for 20 h, and then analyzed for FasL surface expression. (B) 19-BBζ or GD2-28ζ cells (1 × 10^5^) were cultured with SupT1 cells at a 1:1 E:T ratio in the presence of either an isotype control or anti-Fas blocking antibody (1 μg/mL) for either 72 h (left) or 5 h (right), at which point CAR T cell survival or percentage of apoptotic cells (Annexin V^+^ 7AAD^−^) were analyzed, respectively. Six independent donors tested, mean being shown; ns, non-significant, two-way ANOVA. (C) Left: GD2-28ζ cells co-expressing Fas-CD40 were either unstimulated (PBS) or stimulated three times with an immobilized anti-CAR idiotype (anti-Huk666) antibody (1 μg/mL) in the presence of either an isotype control or anti-Fas and anti-FasL antibodies (1 μg/mL per antibody), where CAR T cell counts were measured after each stimulation (counts measured 4 days after each stimulation). Three independent donors tested, error bars are SEM, ∗∗p < 0.01, ∗∗∗p < 0.001, ∗∗∗∗p < 0.0001, two-way ANOVA (statistics comparing isotype control versus anti-Fas/FasL condition upon CAR stimulation). Right: FasL expression of Fas-CD40-GD2-28ζ cells after first round of anti-CAR stimulation. ∗∗p < 0.01, two-tailed paired t test, error bars are SEM.

We observed from the *in vitro* restimulation experiments that co-expression of the Fas-TNFRs augmented both CD19-CAR and GD2-CAR T cell proliferation against target cells not exogenously expressing FasL (Figures 3B, 4C, and S7G). Staining for surface FasL expression in SupT1 and Nalm6 cells revealed that they did not express FasL, even in the presence of IFN-γ (Figures 5A and S8). Moreover, SupT1 cells cultured with 19-BBζ or GD2-28ζ cells did not induce FasL-mediated CAR T cell apoptosis, further evidence that SupT1 cells did not express FasL (Figure 5B). This indicated that tumor-derived FasL was not the only source of FasL in these co-cultures. We therefore hypothesized that T cell-derived FasL upregulated upon activation could be responsible. To address this, Fas-CD40-GD2-28ζ cells were serially stimulated with an anti-CAR idiotype antibody in the presence of anti-Fas and anti-FasL blocking antibodies. As expected, addition of anti-Fas/FasL antibodies significantly decreased Fas-CD40-GD2-28ζ cell proliferation upon CAR activation, with CAR T cell FasL upregulation confirmed (Figure 5C).

### Fas-CD40 significantly enhances 19-BBζ-mediated anti-tumor responses *in vivo*

**Figure 6:**
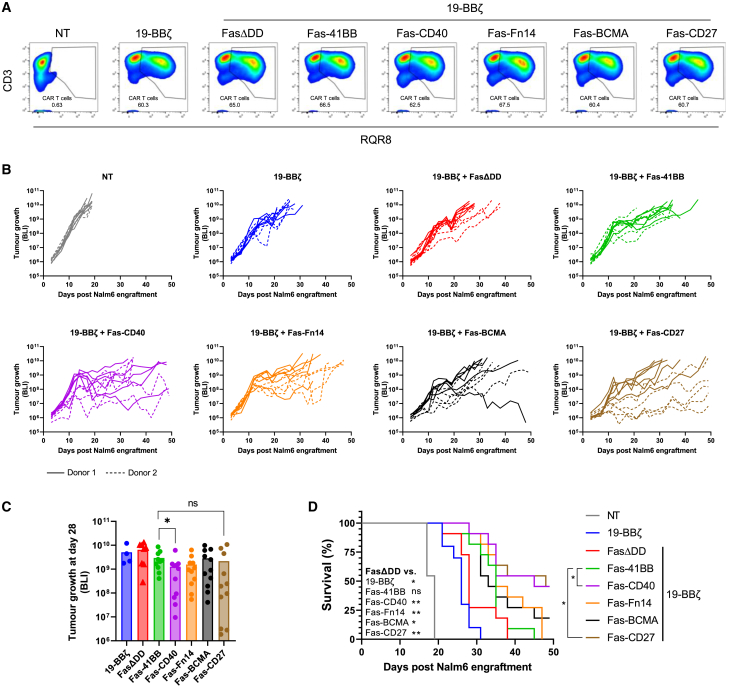
Fas-CD40 is superior for enhancing 19-BBζ-mediated anti-tumor killing and survival *in vivo* (A) Representative flow cytometry plots from one human donor displaying transduction percentages of 19-BBζ cells at day of intravenous (i.v.) injection. (B) NSG mice were administered with 0.5 × 10^6^ Nalm6 cells expressing firefly luciferase (FLuc) by i.v. injection, engrafted for 4 days, and then 3 × 10^6^ CAR T cells (or equivalent total NT cells) were administered by i.v. injection in the tail vein (*n* = 11 mice per cohort, pooled from two independent studies). Tumor growth was measured three times weekly by bioluminescence readout. Individual mouse tumor growths per cohort being shown. (C) Tumor growths from (B) at day 28 post Nalm6 engraftment. Mann-Whitney U test (two-tailed), ∗p < 0.05; ns, non-significant. (D) Kaplan-Meier curve from data shown in (B) showing overall mouse survival. Mantel-Cox test, ∗p < 0.05, ∗∗p < 0.01; ns, non-significant.

From our *in vitro* restimulation experiments with 19-BBζ it was not clear which Fas-TNFR provided the greatest co-stimulatory advantage, as Fas-CD40, Fas-BCMA, and Fas-CD27 all exhibited similar efficacies (Figure 3B). Therefore, we continued our investigations *in vivo* using the xenograft Nalm6 model in NOD-*scid-*IL2Rgamma^null^ (NSG) mice. T cells from two human donors were transduced to express 19-BBζ alone or co-express FasΔDD or the Fas-TNFRs (Figure 6A). Co-expression of Fas-CD40 displayed greatest tumor killing out of all the Fas-TNFRs, with Fas-CD40-19-BBζ-treated mice having significantly lower tumor burden compared with Fas-41BB-19-BBζ (p = 0.0295) (Figures 6B and 6C). Furthermore, co-expression of Fas-CD40 and Fas-CD27 significantly improved mouse survival relative to Fas-41BB (p = 0.0145 and p = 0.0228, respectively) (Figure 6D). There was no significant survival advantage between FasΔDD and Fas-41BB treatments.

## Discussion

The Fas receptor is ubiquitously expressed in T cells and its activation upon binding FasL triggers apoptosis.^4^^,^^5^^,^^6^^,^^7^ Many cancer cells express FasL, in addition to TME cells such as MDSCs, CAFs, Tregs, and the tumor endothelium,^5^^,^^6^ as well as T cells themselves.^10^^,^^11^ Therefore, the Fas/FasL checkpoint may inhibit cancer immunotherapeutic approaches such as adoptive cell therapy by limiting the persistence of T cells.

Strategies to overcome the Fas/FasL checkpoint include systemic antibody blockade.^12^^,^^13^^,^^14^ Approaches applicable to adoptive immunotherapy also include genetic manipulation by *FAS* knockdown and knockout using siRNA and CRISPR-Cas9, respectively.^15^^,^^16^ Additional approaches include the expression of non-functional Fas, such as FasΔDD and Fas-41BB. Both FasΔDD and Fas-41BB rescue T cells from FasL-mediated kill, where the Fas-41BB chimera has the additional advantage of transmitting co-stimulatory signals upon FasL binding.^5^^,^^17^^,^^18^

Different TNFRs may transmit qualitatively different signals due to alternative TRAF recruitment to the TNFR. We hypothesized that chimeric Fas-TNFRs with a different TNFR endodomain to 4-1BB might have different biological effects and hence may be able to better augment immunotherapeutic approaches such as CAR T cells. We generated a library of 17 Fas-TNFR chimeras, identifying Fas-CD40, Fas-CD27, Fas-BCMA, Fas-Fn14, Fas-41BB, Fas-HVEM, and Fas-BAFFR chimeras that could induce T cell proliferation upon binding FasL, with Fas-CD40 eliciting greatest proliferation.

Transcriptional profiling of the five most functional chimeras identified two clusters: (1) Fas-CD27 and Fas-41BB and (2) Fas-CD40, Fas-BCMA, and Fas-Fn14; with cluster 2 displaying greater upregulated DEGs relating to the cell cycle, chemokine and interleukin signaling, JAK-STAT, MAPK/PI3K, and NF-κB pathways, and metabolism. Notably, Fas-CD40 upregulated chemokine receptor/ligand genes: *CCR8*, *CXCR3*, *CXCR4*, *CCL1*, *CXCL10*, and *CXCL13*; which were confirmed at the protein level and have all been implicated in T cell trafficking and could facilitate T cell homing to tumors.^24^^,^^25^^,^^26^^,^^27^ We also observed a trend for Fas-CD40 upregulating *CCL3*, *CCL4*, and *CCL5* transcription; however, this did not reach statistical significance. Interestingly, CCR8 overexpression in CAR T cells enhanced tumor homing, driven by a feedforward loop of activated CAR T cells secreting CCL1 (the cognate ligand for CCR8).^24^ Expression of the Fas-TNFRs increased 19-BBζ-mediated *in vitro* serial cytotoxicity over FasΔDD, except for Fas-41BB, against FasL-expressing targets, with Fas-CD40 inducing greatest proliferation upon serial target stimulation. Furthermore, we showed that Fas-CD40, Fas-Fn14, Fas-BCMA, and Fas-CD27 enhanced 19-BBζ efficacy *in vivo* compared with FasΔDD, with Fas-CD40 demonstrating a significant benefit over Fas-41BB. There was no significant survival advantage *in vivo* between FasΔDD and Fas-41BB, suggesting that complementary *trans*-acting signaling domains between chimeras enhance CAR T cell efficacy, rather than increasing the amplitude of one signaling pathway. Incorporation of *trans*-acting chimeric receptors/signaling domains to enhance CAR T cell activity has been reported previously.^28^^,^^29^^,^^30^^,^^31^

Enhanced CAR T cell-mediated serial cytotoxicity and proliferation upon Fas-CD40, Fas-BCMA, and Fas-Fn14 expression were confirmed in the context of a CD28-containing CAR (19-28ζ) and a CAR targeting a different cognate antigen (GD2-28ζ). Mechanistically, Fas-CD40 appears to demonstrate an advantage over other Fas-TNFRs by enhancing CAR T cell proliferation and maintaining T cell memory, particularly in the context of a CD28-containing CAR, likely mediated by increased TCF-1 expression^23^ and upregulation of FasL, potentially creating a feedforward activation loop. Upregulated FasL induced by Fas-CD40 stimulation could also enhance CAR-independent cancer cytotoxicity, especially with heterogeneous cancers. Fas-CD40 coupled with 19-28ζ exhibited greater tonic activity compared with 19-BBζ, suggesting crosstalk between signaling pathways. One explanation could be that increased CAR tonic signaling mediated by CD28^32^^,^^33^ induces greater FasL upregulation, triggering a feedforward loop via Fas-CD40:FasL paracrine interactions, an effect further exacerbated by CD40 upregulating FasL. Indeed, Künkele et al. demonstrated that CD28-containing CARs caused activation-induced cell death via upregulated CAR T cell-derived FasL.^10^ Importantly, although, this tonic activity did not persist, and rather than this tonic activity inducing functional exhaustion/dysfunction, the opposite was true with Fas-CD40 maintaining the capacity for serial target killing. Importantly, we did not observe any evidence of autonomous proliferation with Fas-CD40, or with any other Fas-TNFR, co-expressed with either 4-1BB- or CD28-containing CARs.

**Figure 7:**
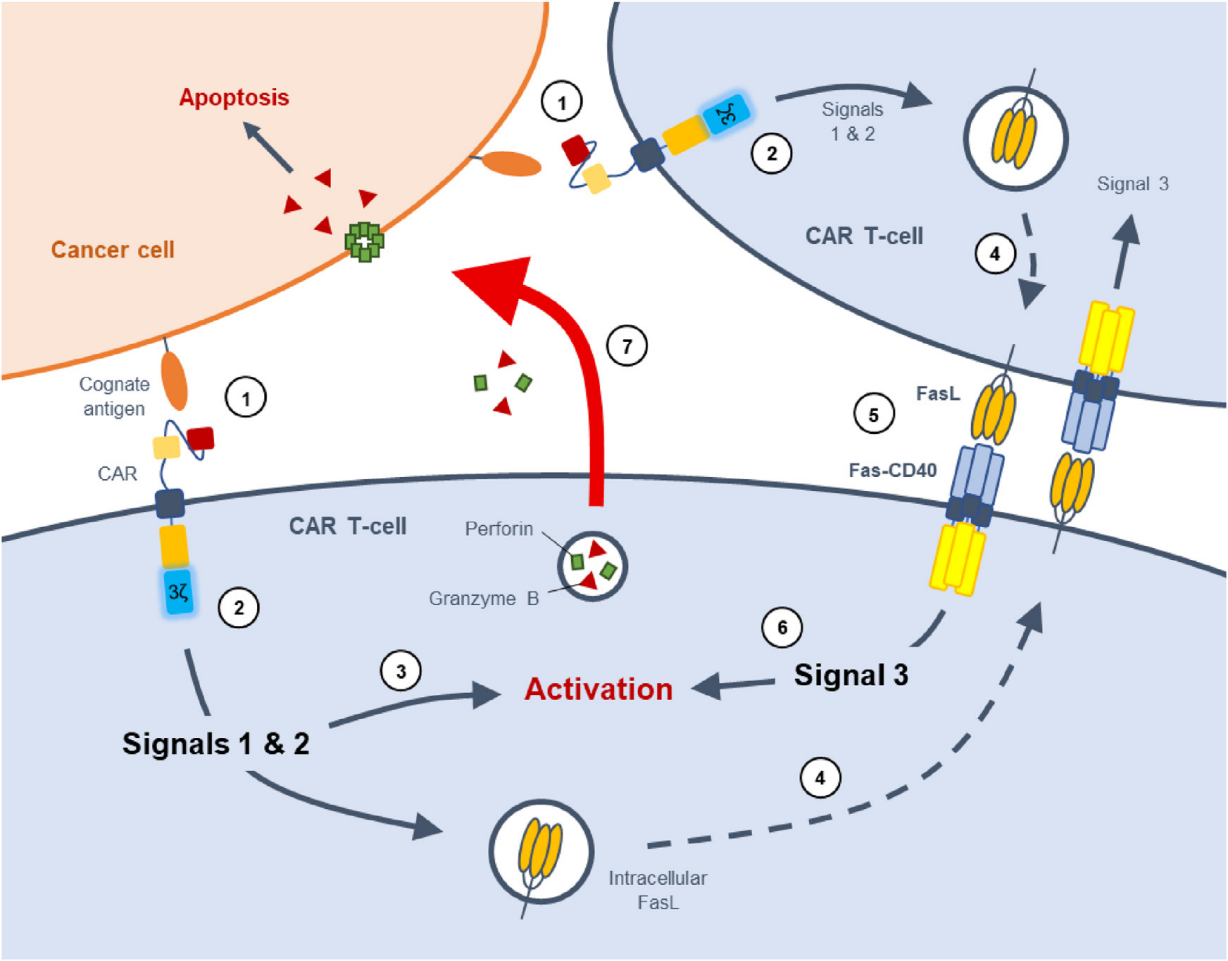
Universal application of Fas-CD40 to enhance CAR T cell efficacy Cartoon illustrating how Fas-CD40 augments CAR T cell efficacy. The CAR binds the cognate antigen on the cancer cell forming an immunological synapse (1). The co-stimulatory domain and CD3ζ within the CAR undergo signal transduction delivering signals 1 and 2 to the cell (2), leading to CAR T cell activation (3). Intracellular stores of FasL are then trafficked to the plasma membrane (4), where Fas-CD40 binds to upregulated FasL on neighboring CAR T cells, as well as FasL-positive cancer cells (5), delivering signal 3 to the cell, augmenting CAR T cell activation (6). Upon activation, the CAR T cell induces target cell killing (7).

Interestingly, we observed the Fas-TNFRs enhanced CAR T cell proliferation and anti-tumor cytotoxicity even when we did not enforce FasL expression on target cells. We subsequently demonstrated that an additional source of FasL for Fas-TNFR activation derives from CAR T cells themselves, an effect observed with TCR-engineered Fas-41BB cells.^17^^,^^18^ Expression of the Fas-TNFRs therefore creates a self-regulatable way to augment CAR T cell activation, irrespective of tumor FasL expression, whereby CAR activation (signals one and two) upregulates FasL surface expression, binding the Fas-TNFR on a sister CAR T cell, which delivers an additional third signal to the CAR T cell (Figure 7). This is akin to physiological TCR-mediated activation between a T cell and an antigen-presenting cell (APC), with APCs delivering additional signals to T cells via presentation of TNFR ligands, a concept explored with expression of full-length 4-1BB or OX40 in CAR T cells, which enhances their efficacy.^34^^,^^35^ However, it remains to be determined whether CAR T cells would have the ability to physically interact with each other within the complex TME to mediate this effect.

As well as the chimeras highlighted above, some chimeras exhibited different effects on T cell function. Fas-LTβR and Fas-CD30 induced constitutive IFN-γ secretion; however, they could not rescue FasL-mediated kill. Expression of full-length LTβR in T cells has been shown to potentiate TCR-activated IFN-γ secretion^36^^,^^37^; however, it did not constitutively induce IFN-γ, therefore the constitutive IFN-γ secretion observed with Fas-LTβR suggests the Fas ecto- and transmembrane domains might be clustering the chimera to form dimers, as has been described previously for Fas prior to ligand binding.^38^ Fas-RANK induced strong constitutive activation of NF-κB, an effect observed with overexpressing full-length RANK^39^; however, this did not correlate with an enhancement of proliferation or IFN-γ secretion. Fas-DcR2 remarkably induced very high levels of NF-κB activation upon binding FasL, consistent with the literature that DcR2 activates NF-κB^21^; however, this did not correlate with increased proliferation or IFN-γ release.

Differences in functional activity between Fas-TNFRs are likely due to qualitative differences in TRAF recruitment. For example, CD40 and BCMA recruits TRAFs 1–3, 5, and 6 upon activation; whereas 4-1BB only recruits TRAFs 1–3.^8^ However, this cannot solely explain the differences in Fas-TNFR performance, as OX40 and RANK, which did not induce proliferation upon binding FasL, also interact with TRAFs 1–3, 5, and 6.^8^ Quantitative differences in the amount of recruited TRAFs to each TNFR will likely also dictate the amplitude of signaling output. TRAF6 could likely be responsible for differentiating between Fas-TNFR function, as TRAF6 appears to be the predominant TRAF for CD40-induced NF-κB activation in dendritic cells,^40^ and TRAF6 also binds BCMA and Fn14, the chimeras of which augmented CAR T cell activity akin to Fas-CD40. TRAF6 is unique from the other TRAFs in several ways: having a different binding motif (P-x-E-x-x-[acidic/aromatic residue]); being involved beyond TNFR signaling such as IL-1R and Toll-like receptor signaling^41^; and being able to activate the Src-family tyrosine kinases resulting in Akt activation via PI3K, in addition to activation of transcription factors NF-κB and AP-1, the latter of which being common among other TRAFs.^41^^,^^42^ Fas-TNFR co-expression with 4-1BB- or CD28-containing CARs adds a further layer of signaling complexity, particularly because CD28 belongs to the immunoglobulin superfamily and as such recruits different signaling proteins to TNFRs, namely SH2 and SH3 domain-containing proteins such as Grb2, PI3K, and Lck.^43^^,^^44^^,^^45^^,^^46^

Protein expression of the Fas-TNFR chimera does not appear to determine its co-stimulatory activity, as Fas-CD27 consistently had the highest expression across multiple CAR architectures; however, its ability to augment CAR T cell activation varied depending on the CAR co-stimulatory domain. Similarly, Fas-BCMA, which had relatively low expression, was able to enhance CAR T cell activation akin to Fas-CD40 with either 4-1BB- or CD28-containing CARs. It is possible that even a low level of Fas-TNFR expression will saturate the amount of available endogenous TRAFs. Fas-TNFR expression does seem particularly important for rescuing FasL-mediated kill, however.

CD40 is typically expressed in APCs such as macrophages, B cells, and dendritic cells, interacting with CD40 ligand on T cells, functioning in a co-stimulatory manner known to activate both canonical and non-canonical NF-κB pathways.^47^^,^^48^ However, CD40 is also expressed in T cells, similarly functioning in a co-stimulatory manner: activating canonical and non-canonical NF-κB pathways, AP-1, and the AP-1 activator JNK^49^; generating T cell memory and ameliorating exhaustion.^50^^,^^51^ CD40 has been identified in several independent screens for enhancing T cell function^37^^,^^52^; and has been synthetically incorporated into CAR T cells, either as a separate module or incorporated into the CAR architecture, displaying superior anti-tumor activity compared with conventional CAR T cells, facilitated by enhanced proliferation and maintaining T cell stemness/memory.^30^^,^^53^^,^^54^^,^^55^^,^^56^^,^^57^^,^^58^ BCMA is expressed in mature B lymphocytes and has been synthetically expressed in CAR T cells, augmenting proliferation^58^; whereas Fn14, expressed in healthy tissue and particularly in solid tumors such as glioblastoma,^59^ has not been previously synthetically expressed in T cells to alter their function. CD27 is a well-known T cell co-stimulatory protein, enhancing T cell function and generating memory.^37^^,^^60^^,^^61^^,^^62^ CD27 has been incorporated into CARs, displaying equivalent *in vivo* functionality to 4-1BB and CD28.^63^^,^^64^

We have extended possibilities of engineering T cells to be resistant to FasL-mediated apoptosis by showing that chimeras of Fas with a range of TNFR endodomains can have potentially useful biological functions. Fusion proteins such as Fas-CD40 may enhance anti-tumor activity when co-expressed with CARs.

## Materials and methods

### Cell lines

HEK293T (ATCC, CRL-11268) cells were cultured in Iscove’s modified Dulbecco’s medium (Sigma, I3390) supplemented with 10% FBS (Biosera, FB-1058) and 2 mM GlutaMAX-1 (Gibco, 35050). SupT1 (ECACC, 95013123), Nalm6 (DSMZ, ACC 128), Raji (ECACC, 85011429), and Jurkat E6.1 (ECACC, 88042803) cell lines were cultured in RPMI-1640 medium (Sigma, R0883) supplemented with 10% FBS and 2 mM GlutaMAX. Cell lines were cultured at 37°C, 5% CO_2_.

### DNA construct generation

All open reading frames were cloned into the MoMLV-based retroviral genome construct SFG. Linear DNA fragments (gBlocks), encoding codon-optimized open reading frames (GeneArt), were synthesized (IDT) and amplified using Q5 DNA polymerase (NEB, M0491L) and oligonucleotide primers (IDT). The resulting PCR products were fractionated on an agarose gel, purified using the QIAquick gel extraction kit (QIAGEN, 28706), and digested with *Esp3I* or *BsaI*-HF v.2 (NEB, R0734L and R3733L, respectively). Digested DNA fragments were purified using the QIAquick PCR purification kit (QIAGEN, 28106) and ligated to gel-purified plasmid backbones using T4 DNA ligase (Roche, 10799009001). New England Biolabs high efficiency competent 5α *E. coli* (NEB, C2987U) were transformed with the ligation reactions, plated onto LB agar containing ampicillin (final concentration of 100 μg/mL), and incubated overnight at 37°C.

Where more than one open reading frame was inserted into the retroviral genome plasmid, self-cleaving peptide sequences derived from *Thosea asigna* virus 2A (T2A), equine rhinitis A virus polyprotein (E2A), or porcine teschovirus-1 2A (P2A) were introduced to facilitate expression from a single mRNA transcript. The suicide/sort marker RQR8 was included in the retroviral genome plasmids to enable detection of transduced cells.^22^

### Retroviral production

HEK293T cells (1.5 × 10^6^) were transiently transfected with an RD114 envelope expression plasmid (RDF, a gift from M. Collins, University College London), a Gag-pol expression plasmid (PeqPam-env, a gift from E. Vanin, Baylor College of Medicine), and the transgene of interest expressed in a retroviral (SFG) vector plasmid at a ratio of 1:1.5:1.5 (total DNA = 12.5 μg). Transfections were performed with GeneJuice (Millipore, 70967) according to the manufacturer’s instructions and viral supernatants were harvested 48 h post transfection and stored at −80°C.

### Calculation of functional retroviral titers

Functional viral titers of retroviral supernatant were calculated using frozen supernatant on primary human T cells activated with TransAct (Miltenyi Biotec, 130-111-160), 10 ng/mL IL-7 (Miltenyi Biotec, 130-095-367) and 10 ng/mL IL-15 (Miltenyi Biotec, 130-095-760) for 48 h. Retroviral supernatant was serially diluted into 24-well tissue culture plates (Corning, 351147) coated with RetroNectin (Takara Bio, T100B), where 3 × 10^5^ activated T cells were seeded and then spun by centrifugation for 1,000 × *g*, 40 min at room temperature, and then cultured at 37°C, 5% CO_2_. Transduced cells were identified by measuring for CAR expression using anti-fmc63 and anti-Huk666 idiotypes (both produced in-house). Viral titers were calculated with T cells that were less than 20% transduced.

### Transduction of primary human T cells and cancer cell lines

Peripheral blood mononuclear cells (PBMCs) were isolated from whole human blood (NHS Blood and Transplant) by density centrifugation with Ficoll-Paque Plus (GE-Healthcare, GE17-1440-03) according to the manufacturer’s instructions. Isolated PBMCs were activated with TransAct and 10 ng/mL IL-7 and IL-15 and cultured in RPMI-1640 supplemented with 10% FBS and 2 mM GlutaMAX and cultured at 37°C, 5% CO_2_. At 48 h post activation, PBMCs (1 × 10^6^) were seeded onto RetroNectin-coated 6-well tissue culture plates (Corning, 351146) with retroviral vector and spun by centrifugation for 1,000 × *g*, 40 min at room temperature, and then cultured at 37°C, 5% CO_2_. PBMCs were transduced at equal multiplicity of infections (MOIs) across all cohorts. SupT1 and Nalm6 cell lines were transduced in a similar manner to PBMCs, without activation with TransAct and IL-7/IL-15 and using non-titered retroviral supernatant. Expression of the transgene in PBMCs and cancer cell lines was assessed 72 h post transduction by flow cytometry. RQR8 expression was detected using an anti-CD34 antibody.

### Flow cytometry and antibodies

Flow cytometry was performed using MACSQuant 10 and X flow cytometers (Miltenyi Biotec). All staining, unless specified otherwise, was performed at room temperature for 10 min, protected from light, with antibodies diluted in either PBS (Sigma, D8537) or cell staining buffer (BioLegend, 420201). Cell viability dyes used were 7-AAD (BioLegend, 420404) or Sytox blue (Thermo Fisher Scientific, S34857). To detect TCF-1 expression, cells were first surface stained for RQR8 as described above and then stained for TCF-1 using the True-Nuclear Transcription Factor Buffer Set (BioLegend, 424401).

Antibodies were from BioLegend, unless otherwise stated. Antibodies used were: CD2-PE (300208), CD3-PE Cy7 (344816), CD34-APC (R&D Systems, FAB7227A), TCF-1-PE (655208), GD2-APC (357306), FasL-BV421 (306412), Fas-PE (305608), Fas-APC Cy7 (305636), CCR8-PE (360604), ICOSL-PE (309404), ICOS-PE (313508), CD45RA-PE Texas Red (Invitrogen, MHCD45RA17), CD62L-Pacific blue (304826), LAG3-FITC (369308), PD-1-PE (329906), TIM3-BV421 (345008), CD8-APC Cy7 (301016), and PE Mouse IgG1 Isotype Control (981804). Anti-CD19 and anti-GD2 CARs were detected using anti-Fmc63 and anti-Huk666 idiotypes, respectively (produced in-house), and anti-mouse IgG secondary antibody conjugated to Alexa Fluor 647 (Jackson ImmunoResearch, 115-605-071).

Antibodies used for western blotting were from Cell Signaling Technology. The primary antibodies (sourced from rabbits) used were p-ERK (9101), ERK (4695), p-p38 (4511), p38 (8690), p-JNK (4668), JNK (9252), PI3K (3011), STAT1 (14994), STAT3 (4904), STAT5 (94205), and GAPDH (5174). The secondary antibody was an anti-rabbit IgG horseradish peroxidase (HRP)-linked antibody (7074).

### Generation of antigen-expressing cell lines and reporter cell lines

For the generation of FasL-expressing cell lines, SupT1 and Nalm6 cell lines were nucleofected (Lonza) with Cas9 ribonucleic protein (RNP) complexes in SF buffer (Lonza), using the pulse codes CM-150 or CV-104, respectively. RNP complexes were formed using 50 pmol of Alt-R S.p. HiFi Cas9 Nuclease V3 endonuclease (IDT, 1081060) and 100 pmol of the following single-guide RNAs (sgRNAs) (Synthego) targeting the human *FAS* locus; sgRNA 1: ggaguugaugucagucacuu; sgRNA 2: gugacugacaucaacuccaa; sgRNA 3: ugacaucaacuccaagggau; sgRNA 4: cuuccucaauuccaaucccu. Knockout (KO) efficiency was determined by flow cytometry, staining for Fas expression. Non-electroporated Fas^+^ cells were eliminated after addition of 100 ng/mL *Mega*FasL (AdipoGen, AG-40B-0130-3010) for 48 h. SupT1^FasKO^ and Nalm6^FasKO^ cells were then transduced with retroviral supernatant to express human FasL, where transduction efficiency was measured by flow cytometry staining for FasL.

To produce SupT1 cells expressing human CD19, SupT1 cells were transduced with retroviral supernatant encoding human CD19 and sorted for CD19 expression by fluorescence-activated cell sorting (FACS) on the BD FACSMelody Cell Sorter according to the manufacturer’s instructions. To produce SupT1 cells expressing human GD2 and/or FasL, wild-type SupT1 or SupT1^FasKO^ cells were transduced with retroviral supernatant encoding GD2- and GD3-synthases, separated by a 2A self-cleaving peptide, or also dual-transduced with retroviral supernatant to express FasL, and were then sorted for GD2 and/or FasL expression by FACS on the BD FACSMelody Cell Sorter.

To produce the NF-κB Jurkat reporter cell line, Jurkat E6.1 cells were electroporated by nucleofection (using a platform from Lonza) with a plasmid encoding five copies of an NF-κB response element linked to luciferase (Promega, N1111) and a hygromycin resistance gene. Jurkat cells expressing the NF-κB reporter were cultured under hygromycin selection (100 μg/mL).

### Western blotting

CAR T cells were lysed with RIPA buffer (Merck, 20-188) including a protease and phosphatase inhibitor cocktail (Abcam, ab201119), with NuPAGE LDS sample buffer (Thermo Fisher Scientific, NP0007) and β-mercaptoethanol (Bio-Rad, 1610710) subsequently being added. Lysate samples were heated to 95°C for 5 min and then loaded onto an SDS-polyacrylamide gel (Bio-Rad, 4561096) and resolved at 170 V for approximately 90 min. Proteins were transferred to a Trans-Blot Turbo PVDF membrane (Bio-Rad, 1704157) using the Trans-Blot Turbo Transfer System (Bio-Rad, 1704150) and then blocked in 5% BSA (Merck, A7906) in Tris-buffered saline Tween-20 (TBST) buffer (Thermo Fisher Scientific, 28360) for 1 h at room temperature. Membranes were stained with primary antibodies in 5% BSA in TBST overnight at 4°C, washed in TBST, stained with a secondary HRP-linked antibody in 5% BSA in TBST for 1 h at room temperature, and then washed in TBST. Membranes were treated with HRP substrate (Thermo Fisher Scientific, 11546345) for 3 min and then imaged on an Azure c600 analyser (Azure Biosystems).

### *In vitro* cytotoxicity and proliferation assays

CAR T cells were co-cultured with 5 × 10^4^ target cells (unless stated otherwise) at the stated E:T, where target cells were detected by flow cytometry by the absence of CD2, CD3, and RQR8 expression. Surviving target cells were normalized to surviving target cell numbers in co-cultures with non-transduced (NT) T cells. The number of CAR T cells was quantified using CountBright Counting Beads (Thermo Fisher Scientific, C36995). For the restimulation experiments, CAR T cells were initially co-cultured with 5 × 10^4^ target cells at the stated E:T, and then restimulated with target cells as described, twice weekly for up to a total of 10 target stimulations. Nalm6^FasKO^ and SupT1^FasKO^ parental cell lines were used for the restimulation experiments.

### Detection of cytokines

Cytokine concentrations in cell culture supernatants were measured by ELISA using kits to detect IFN-γ (BioLegend, 430104), IL-2 (BioLegend, 431804), CCL1 (R&D Systems, DY272), CXCL10 (R&D Systems, DY266), and CXCL13 (R&D Systems, DY801) according to the manufacturer’s instructions using a Multiskan FC microplate photometer (Thermo Scientific).

### Detection of apoptotic cells

Transduced T cells were treated as described, incubated for 5 h at 37°C, 5% CO_2_, surface stained for CD3 and RQR8, washed once in PBS, washed once in Annexin V binding buffer (BioLegend, 422201), resuspended in Annexin V binding buffer with Annexin V BV421 (BioLegend, 640924), and incubated for 15 min at room temperature protected from the light. Cells were then washed and resuspended in Annexin V binding buffer containing 7-AAD and analyzed by flow cytometry. Apoptotic cells were defined as being Annexin V^+^ 7-AAD^−^.

### Immobilized FasL assays

Recombinant FasL (2 μg) (PeproTech, 310-03H) was immobilized onto 96-well microplates (Starlab, CC7672-7596) overnight at 4°C and the plate was washed several times with PBS. For the proliferation experiments, 5 × 10^4^ CAR T cells was seeded onto the FasL-immobilized microplate and incubated for 5 days. The number of CAR T cells was quantified by flow cytometry, using CountBright Counting Beads. For the measurement of NF-κB activity, 1 × 10^5^ transduced NF-κB Jurkat reporter cells were seeded onto the FasL-immobilized microplate, incubated overnight, and then treated as described.

### NF-κB reporter assay

Transduced NF-κB Jurkat reporter cells (1 × 10^5^) were cultured with immobilized FasL (20 μg/mL) overnight, at which point cells were analyzed with the Bright-Glo Luciferase Assay System (Promega, E2610) according to the manufacturer’s instructions, and then luminescence measured on a Varioskan LUX microplate reader (Thermo Scientific).

### Transcriptomic analysis using the NanoString platform

Recombinant FasL (2 μg) (PeproTech, 310-03H) was immobilized onto 96-well microplates (Starlab, CC7672-7596) overnight at 4°C and the plate was washed several times with PBS. CAR T cells (2 × 10^5^) were then seeded onto the FasL-immobilized microplate and incubated at 37°C, 5% CO_2_ for 3 days. RNA was extracted from the microplate using the RNAspin Mini Kit (Merck, GE25-0500-71) and quantified using a NanoDrop Spectrophotometer (Thermo Fisher Scientific). Extracted RNA (50 ng) was sequenced using the nCounter CAR-T Characterization Panel (NanoString) and analyzed on the nCounter SPRINT Profiler (NanoString) according to the manufacturer’s instructions.

### Memory and exhaustion phenotyping

For memory phenotyping, CAR T cells were stained for CD62L and CD45RA expression, with CD62L^+^CD45RA^+^ being naive T cells (TN), CD62L^+^CD45RA^−^ being central memory T cells (TCM), CD62L^−^CD45RA^−^ being effector memory T cells (TEM), and CD62L^−^CD45RA^+^ being effector memory T cells expressing CD45RA (TEMRA). For expression of markers associated with exhaustion, CAR T cells were stained for PD-1, LAG3, and TIM3, using Boolean gating to identify cells expressing one, two, or three of these markers. To get an accurate representation of the cell’s phenotype, only cohorts that had at least 2,000 cells acquired in the CAR T gate (CD3^+^RQR8^+^) on the flow cytometer were analyzed. Any cohorts below this threshold were excluded from analysis.

### Immobilized anti-GD2 CAR restimulation assays

Anti-GD2 CAR ideotype antibody (anti-Huk666) (100 ng) was immobilized onto 96-well microplates overnight at 4°C and the plates were washed several times with PBS prior to seeding with 1 × 10^5^ CAR T cells, which were incubated for 3 or 4 days. CAR T cell numbers were enumerated by flow cytometry using CountBright Counting Beads and were reseeded onto another anti-GD2 CAR immobilized microplate.

### *In vivo* studies

All animal studies were performed under a UK Home Office-approved project license. Six- to 10-week-old female NSG mice (Charles River Laboratory) were raised under pathogen-free conditions. Nalm6 cells (0.5 × 10^6^) engineered to express firefly luciferase and an HA tag were inoculated intravenously into NSG mice 4 days prior to CAR T cell engraftment. Mice were randomized 1 day prior to CAR T cell engraftment, where the following day 3 × 10^6^ CAR T cells were injected intravenously. Tumor engraftment and ongoing tumor growth was measured by bioluminescent imaging using the IVIS Spectrum System (PerkinElmer) after intraperitoneal injection of VivoGlo luciferin (Promega, P1041). Human T cells were transduced at an MOI of 1.5.

### Data analysis

Data and statistical analyses were performed on GraphPad Prism 9. Flow cytometry analysis was performed on FlowJo (v.10.8.1). Transcriptomic analysis from the NanoString platform was performed using nSolver 4.0, R, and Python. Quantification of western blot images were performed using ImageJ.

## Data Availability

The data that support the findings of this study are available from the corresponding author upon reasonable request.

## Supplemental information

Document S1. Figures S1–S8

Table S1. Electronic file showing NanoString analysis comparing mRNA transcripts from cells expressing 19-ζ and FasΔDD or Fas-TNFRs versus cells expressing 19-ζ alone (baseline) Experiment performed as described in Figure 1F.

Table S2. Electronic file showing NanoString analysis comparing mRNA transcripts from cells expressing 19-ζ and Fas-TNFRs versus cells expressing 19-ζ and FasΔDD (baseline) Experiment performed as described in Figure 1F.

Document S2. Article plus supplemental information
