# Supplementary material for: Novel Fas-TNFR chimeras that prevent Fas ligand-mediated kill and signal synergistically to enhance CAR T cell efficacy
Source: Mol Ther Nucleic Acids. 2023 Apr 25;32:603–21. doi: 10.1016/j.omtn.2023.04.017 (PMC10185706; doi:10.1016/j.omtn.2023.04.017)
Supplement: Document S1. Figures S1–S8 [file mmc1.pdf]

OMTN, Volume *32*

Supplemental information

Novel Fas-TNFR chimeras that prevent Fas ligand-mediated kill and signal synergistically to enhance CAR T cell efficacy

Callum McKenzie, Mohamed El-Kholy, Farhaan Parekh, Mathew Robson, Katarina Lamb, Christopher Allen, James Sillibourne, Shaun Cordoba, Simon Thomas, and Martin Pule

Figure S1

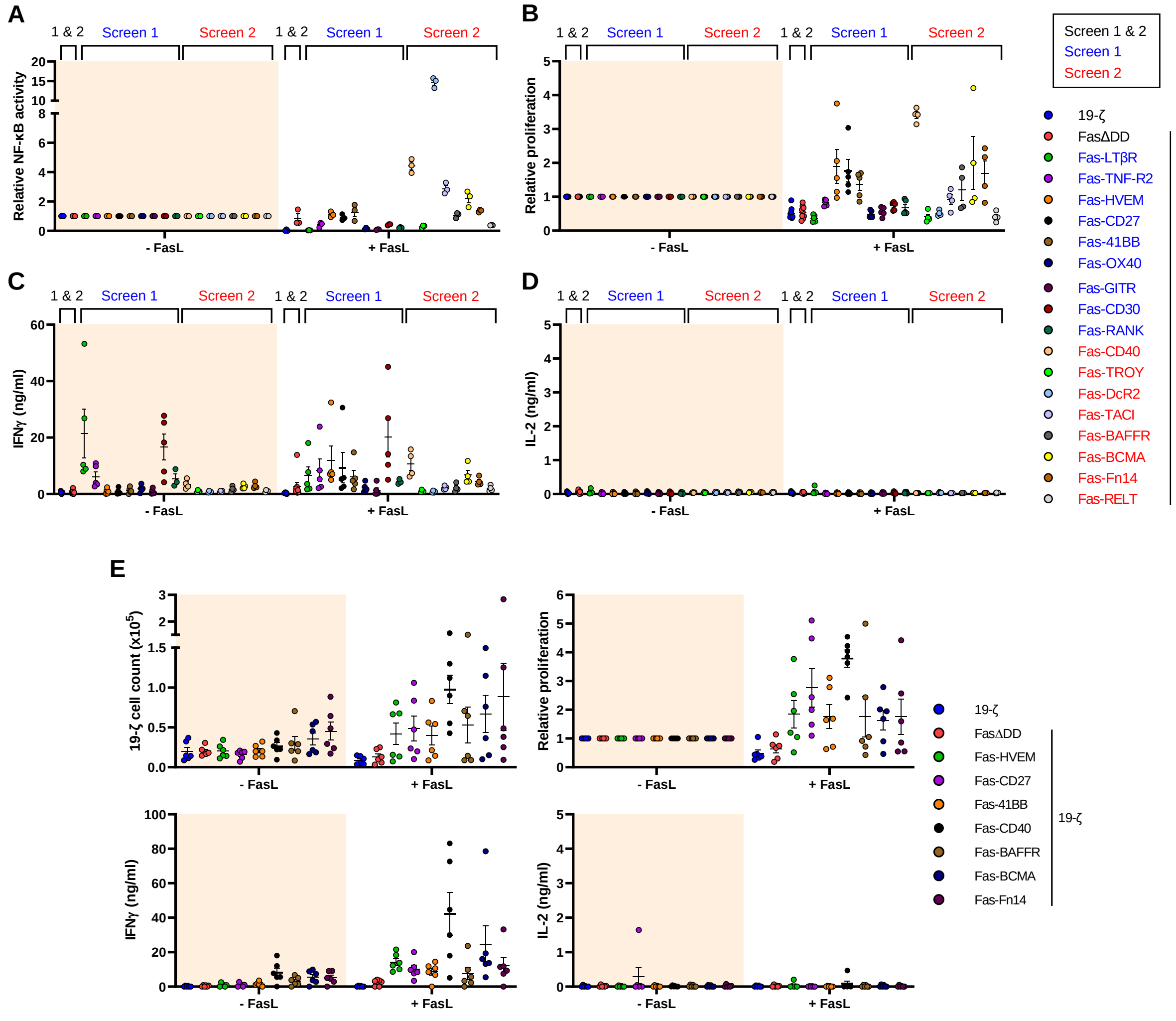

Figure S1. Fas-TNFRs alter CAR T-cell activity. (A) Relative NF-κB activity from Figure 1D. Error bars are SEM. (B) Relative T-cell proliferation from Figure 1E. Error bars are SEM. (C and D) After the five-day timepoint from Figure 1E, cell culture supernatant was analysed for IFNγ (C) and IL-2 (D). Error bars are SEM. (E) Fas-TNFR chimeras that induced proliferation upon binding FasL (Figures 1E, S1B) were tested under identical assay conditions using six different independent donors. 5x10^4 19-ζ cells were cultured with or without immobilised recombinant FasL (20 µg/ml) for five days, where absolute cell counts (top left) and relative cell counts (top right) were measured, along with IFNγ (bottom left) and IL-2 (bottom right) secretion into the cell culture supernatant. Error bars are SEM.

Figure S2

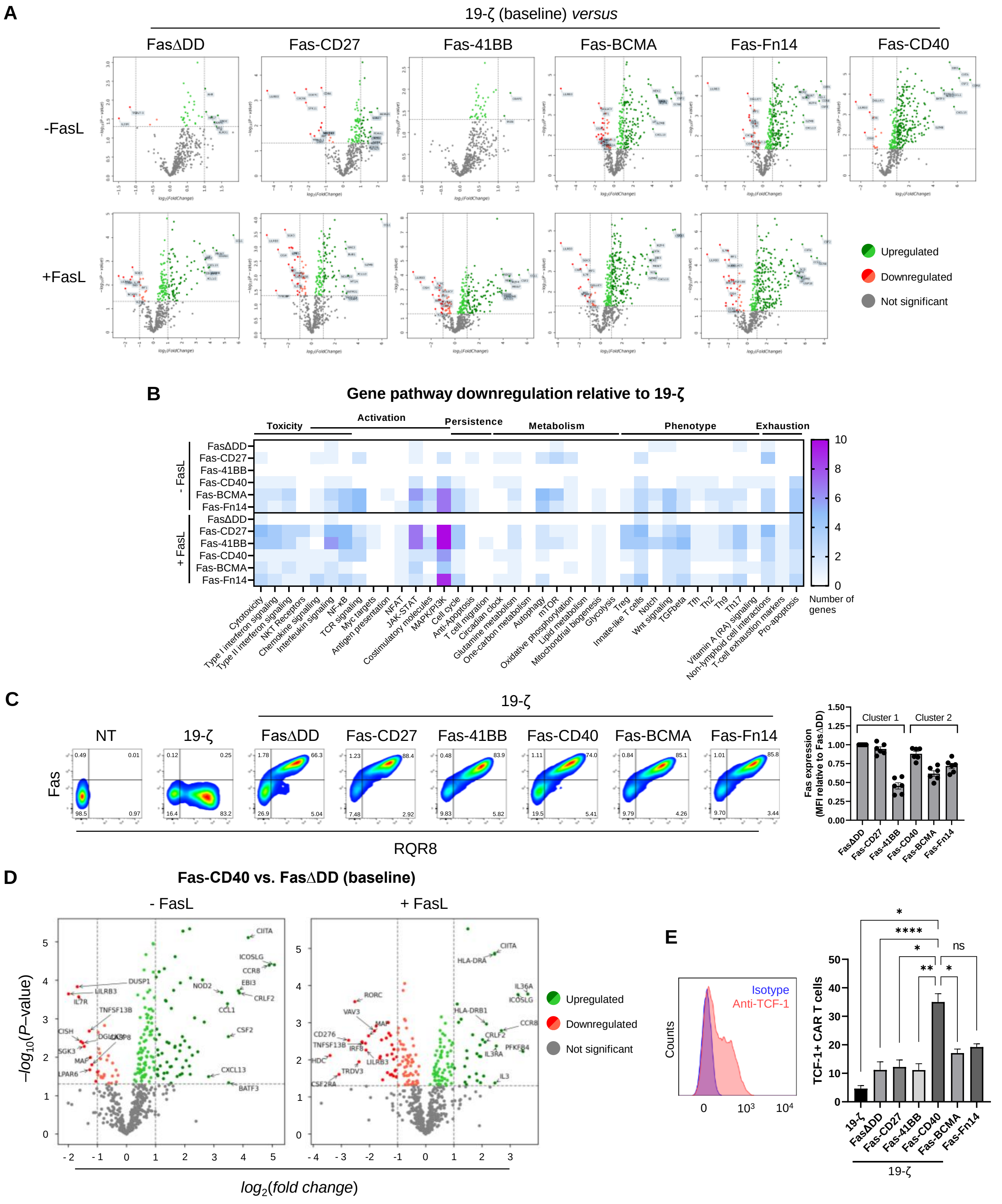

A
19-ζ (baseline) *versus*
FasΔDD
Fas-CD27
Fas-41BB
Fas-BCMA
Fas-Fn14
Fas-CD40
-FasL
+FasL
Upregulated
Downregulated
Not significant
B
Gene pathway downregulation relative to 19-ζ
Toxicity
Activation
Persistence
Metabolism
Phenotype
Exhaustion
- FasL
+ FasL
FasΔDD
Fas-CD27
Fas-41BB
Fas-CD40
Fas-BCMA
Fas-Fn14
Number of genes
Cytotoxicity
Type I interferon signaling
Type II interferon signaling
NKT Receptors
Chemokine signalling
Interleukin signalling
NF-κB
TCR signaling
Myc targets
Antigen presentation
NFAT
JAK-STAT
Costimulatory molecules
MAPK/PI3K
Cell cycle
Anti-Apoptosis
T cell migration
Circadian clock
Glutamine metabolism
One-carbon metabolism
Autophagy
mTOR
Oxidative phosphorylation
Lipid metabolism
Mitochondrial biogenesis
Glycolysis
Treg
Innate-like T cells
Notch
Wnt signaling
TGFbeta
Tfh
Th2
Th9
Th17
Vitamin A (RA) signaling
Non-lymphoid cell interactions
T-cell exhaustion markers
Pro-apoptosis
C
19-ζ
NT
19-ζ
FasΔDD
Fas-CD27
Fas-41BB
Fas-CD40
Fas-BCMA
Fas-Fn14
Fas
RQR8
Fas expression (MFI relative to FasΔDD)
Cluster 1
Cluster 2
D
Fas-CD40 vs. FasΔDD (baseline)
- FasL
+ FasL
−log10(P−value)
log2(fold change)
Upregulated
Downregulated
Not significant
E
Isotype
Anti-TCF-1
Counts
TCF-1+ CAR T cells
19-ζ
FasΔDD
Fas-CD27
Fas-41BB
Fas-CD40
Fas-BCMA
Fas-Fn14
ns

Figure S2. Fas-TNFRs alter T-cell transcriptome. (A) Volcano plots of 19-ζ cells co-expressing either FasΔDD or the stated Fas-TNFRs compared to 19-ζ alone, in the presence or absence of immobilised FasL (as described in Figure 1F). (B) Number of significantly ($P < 0.05$) downregulated DEGs relative to 19-ζ were categorised by pathway involvement. (C) Left: Representative flow cytometry plots from one human T-cell donor transduced to express either 19-ζ alone or 19-ζ co-expressing FasΔDD or the stated Fas-TNFRs. Right: median fluorescence intensity (MFI) of the Fas-TNFRs relative to FasΔDD MFI, measured from top right quadrant in flow cytometry plots. Six independent donors tested. (D) Volcano plot from experiment described in Figure 1F of Fas-CD40-19-ζ cells compared to FasΔDD-19-ζ cells with or without FasL incubation. (E) Left: representative flow cytometry plot from one donor stained for TCF-1 expression with an isotype control. Right: 19-ζ cells were incubated with *Mega*FasL (10 ng/ml) for 48 hours and then stained for TCF-1. Three independent donors, error bars are SEM.

Figure S3

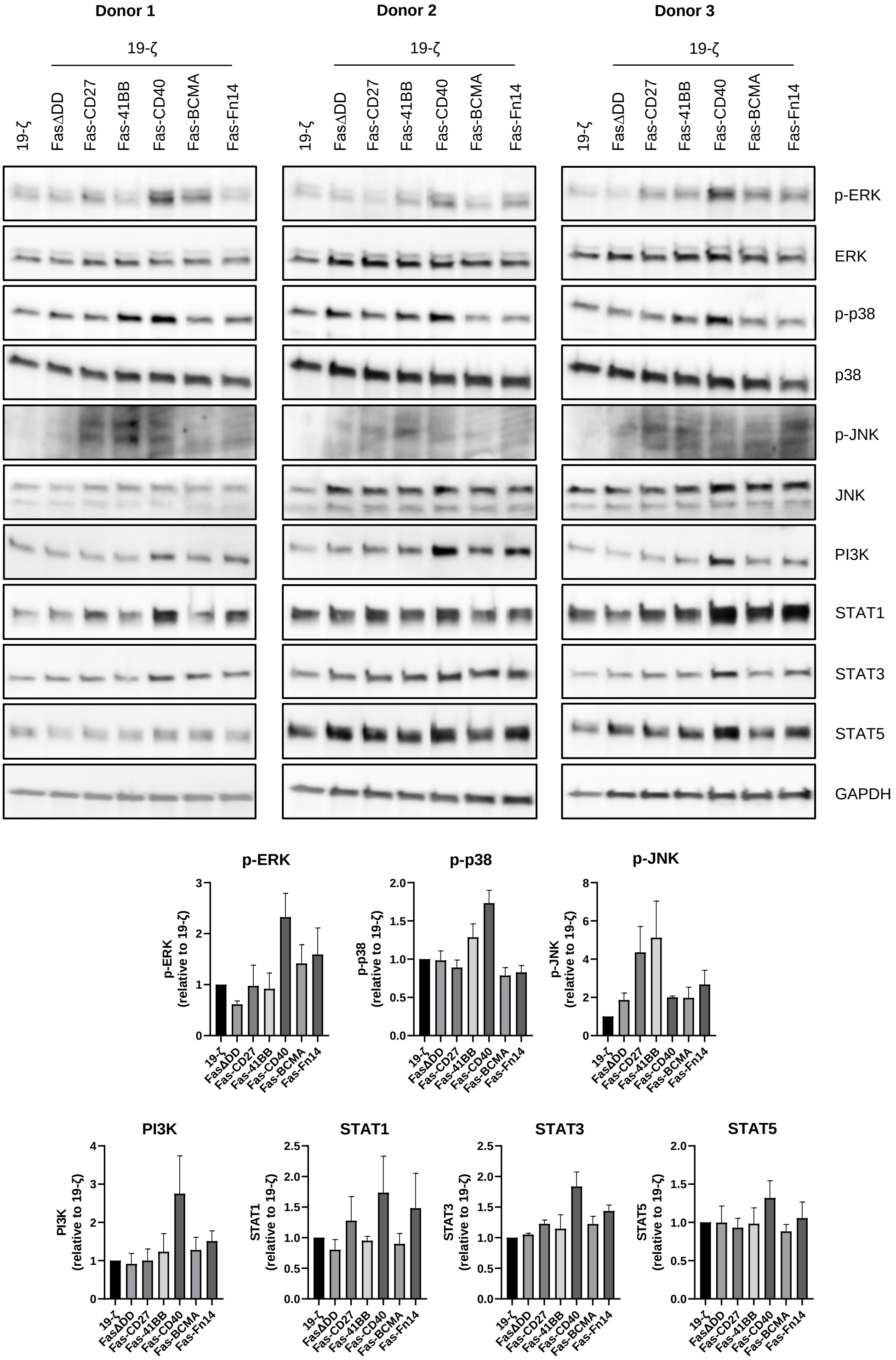

Figure S3. Fas-CD40 activates ERK and p38, and upregulates PI3K and STAT protein expression. Top: Western blots of 19-ζ cells expressing FasΔDD or stated Fas-TNFRs. Three independent donors tested. Bottom: Quantification of phosphorylated ERK, p38, and JNK; and PI3K, STAT1, STAT3 and STAT5 expression from Western blots shown above. Phosphorylation was calculated by normalising phosphorylated protein signal to total (non-phosphorylated) protein and then made relative to 19-ζ alone. Protein expression of PI3K and STAT proteins were calculated by normalising to GAPDH loading control and then made relative to 19-ζ alone. Error bars are SEM.

Figure S4

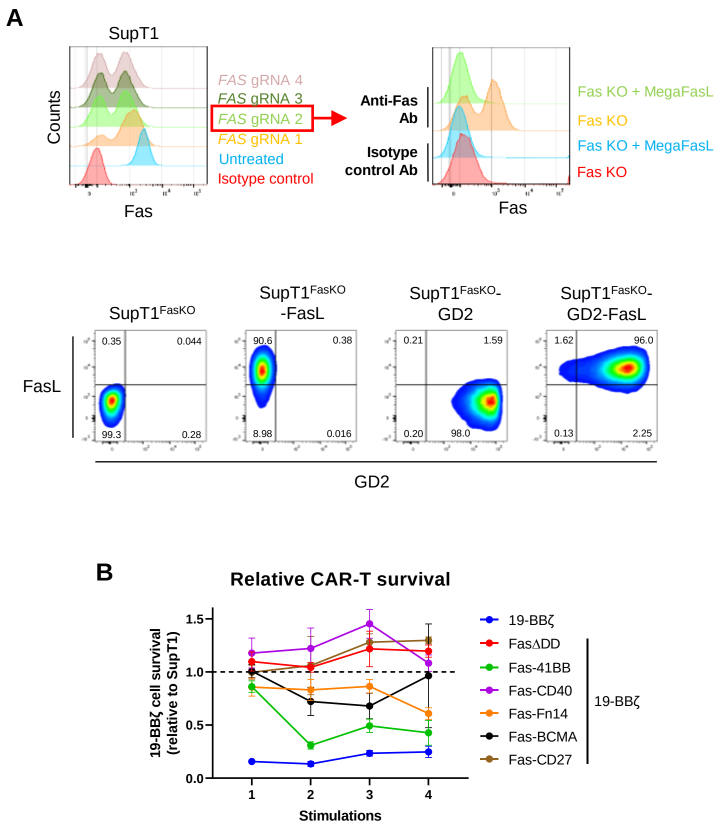

Figure S4. Generation of SupT1 target cells expressing FasL and GD2. (A) Top: SupT1 cells were transfected by nucleofection with Cas9 nuclease and four guide RNAs (gRNA) targeting *FAS*, where knockout (KO) efficiency was determined by staining for surface Fas expression. SupT1 cells treated with *FAS* gRNA 2 were then treated with *Mega*FasL (100 ng/ml) to eliminate non-transfected Fas positive cells to create a pure Fas KO population. Bottom: SupT1^{FasKO} cells were transduced to express FasL, GD2 or FasL and GD2. (B) Relative 19-BBζ cell survival after four target cell stimulations from Figure 2E. Error bars are SEM.

Figure S5

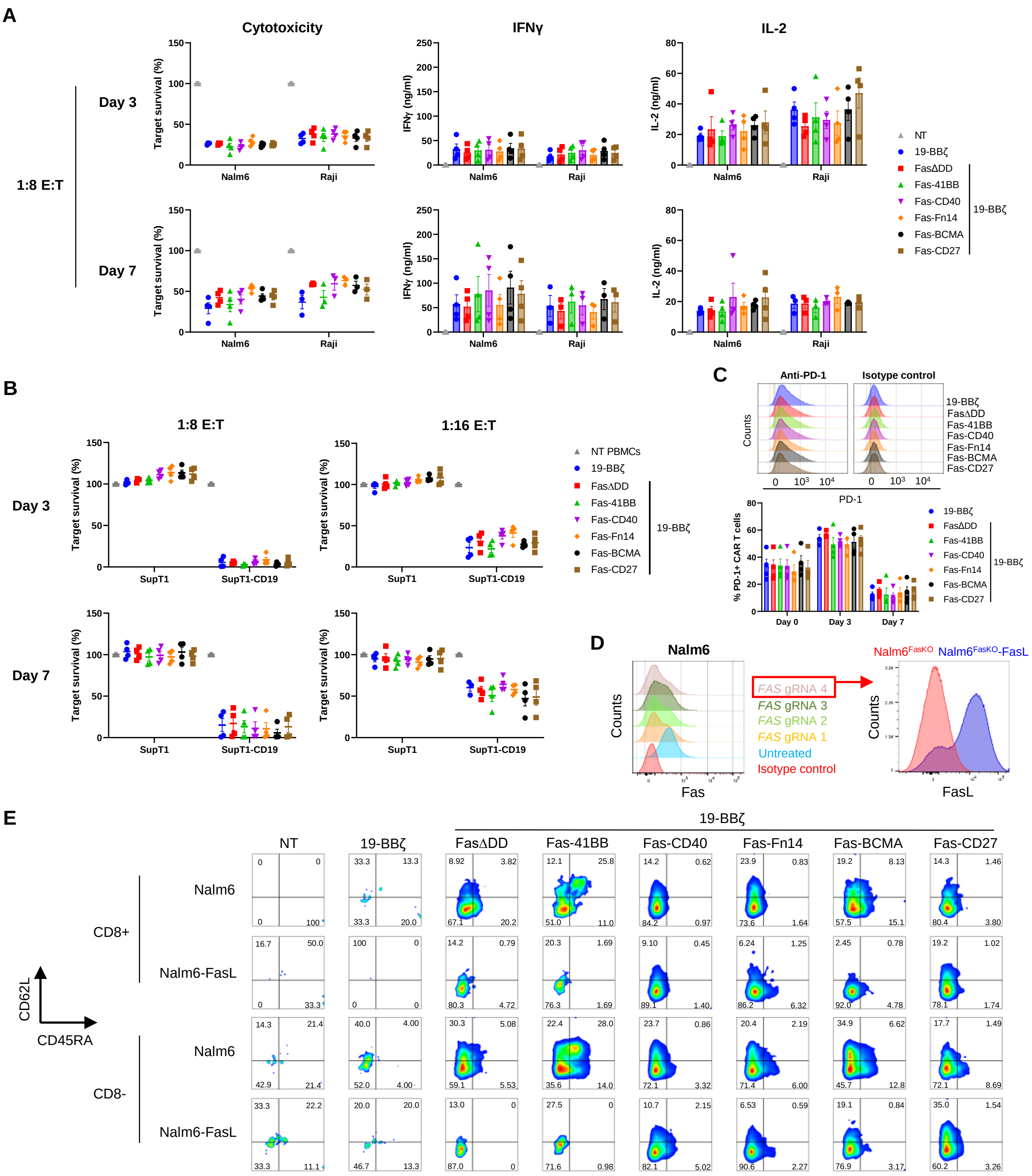
A
Cytotoxicity
IFNγ
IL-2
1:8 E:T
Day 3
Day 7
Target survival (%)
IFNγ (ng/ml)
IL-2 (ng/ml)
Nalm6
Raji
NT
19-BBζ
FasΔDD
Fas-41BB
Fas-CD40
Fas-Fn14
Fas-BCMA
Fas-CD27
19-BBζ
B
1:8 E:T
1:16 E:T
Day 3
Day 7
Target survival (%)
SupT1
SupT1-CD19
NT PBMCs
19-BBζ
FasΔDD
Fas-41BB
Fas-CD40
Fas-Fn14
Fas-BCMA
Fas-CD27
19-BBζ
C
Anti-PD-1
Isotype control
Counts
PD-1
19-BBζ
FasΔDD
Fas-41BB
Fas-CD40
Fas-Fn14
Fas-BCMA
Fas-CD27
% PD-1+ CAR T cells
Day 0
Day 3
Day 7
19-BBζ
D
Nalm6
Counts
Fas
FAS gRNA 4
FAS gRNA 3
FAS gRNA 2
FAS gRNA 1
Untreated
Isotype control
Nalm6FasKO Nalm6FasKO-FasL
Counts
FasL
E
19-BBζ
NT
19-BBζ
FasΔDD
Fas-41BB
Fas-CD40
Fas-Fn14
Fas-BCMA
Fas-CD27
CD8+
CD8-
Nalm6
Nalm6-FasL
CD62L
CD45RA

Figure S5. Fas-TNFRs display equivalent target cytotoxicity after one round of stimulation. (A) Same experiment as described in Figure 3A, however cells were cultured at a 1:8 E:T. (B) 19-BBζ cells co-expressing FasΔDD or the Fas-TNFRs were cultured with SupT1 or SupT1-CD19 cells for three or seven days, at 1:8 and 1:16 E:Ts, measuring for target survival. Four independent donors tested, error bars are SEM. (C) Top: representative flow cytometry plots from one donor showing PD-1 expression on CAR T cells without target stimulation (day 0). Bottom: Graph showing CAR T-cell PD-1 expression at day 0, 3 and 7 of Nalm6 co-culture (1:4 E:T). Four donors, error bars are SEM. (D) Left: Nalm6 cells were transfected by nucleofection with Cas9 nuclease and four guide RNAs (gRNA) targeting *FAS*, where KO efficiency was determined by staining for surface Fas expression. Right: Nalm6 cells treated with *FAS* gRNA 4 were then treated with *Mega*FasL (100 ng/ml) to eliminate non-transfected Fas positive cells to create a pure Fas KO population ($Nalm6^{FasKO}$) and were then transduced to express FasL ($Nalm6^{FasKO}$-FasL). (E) Representative flow cytometry plots from one donor showing memory phenotype from data in Figure 3E.

Figure S6

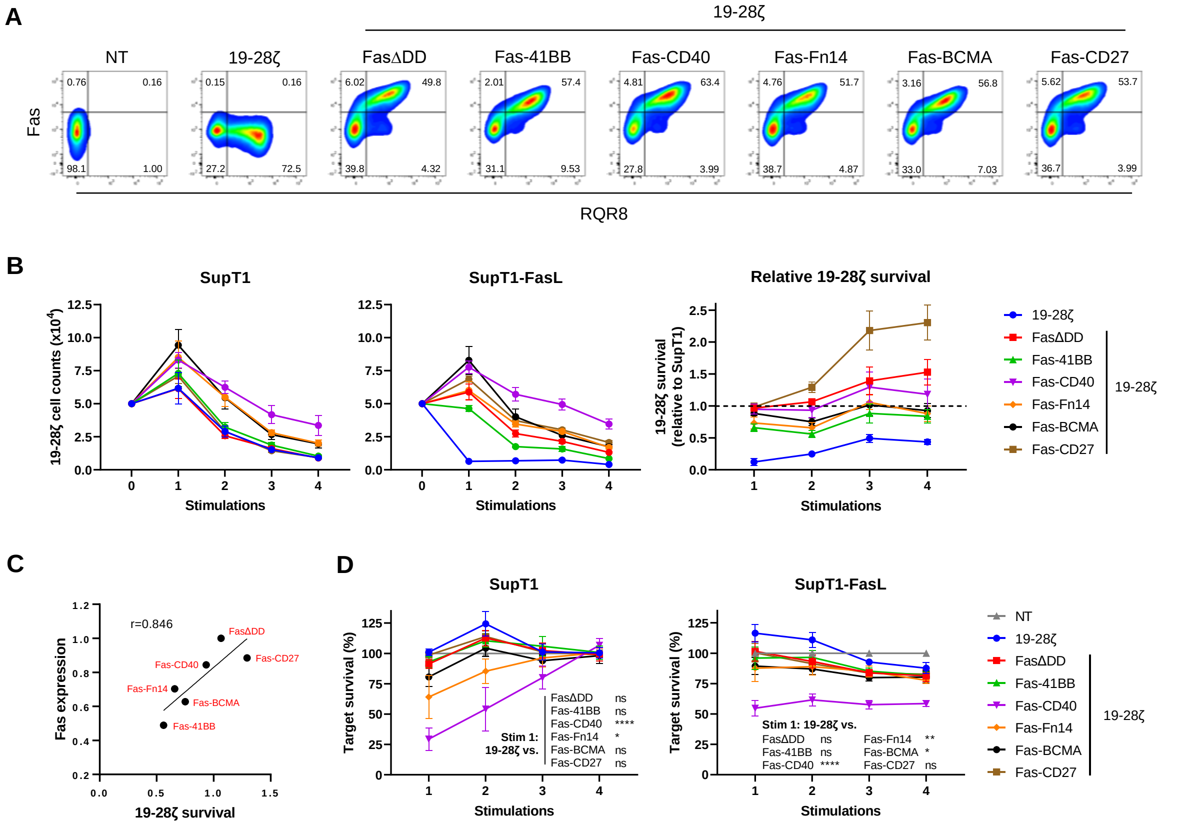

Figure S6. Fas-CD40 co-expressed with 19-28ζ increases background cytotoxicity. (A) Representative flow cytometry plots from one human T-cell donor transduced to express either 19-28ζ alone or co-express FasΔDD or the stated Fas-TNFRs. (B) Left and middle: 19-28ζ cells co-expressing FasΔDD or the Fas-TNFRs from four independent donors were stimulated with $5x10^4$ $SupT1^{FasKO}$ or $SupT1^{FasKO}$-FasL cells up to four times, at an initial 1:1 E:T, with cell counts being analysed after each stimulation. Right: Relative 19-28ζ cell survival from the four target stimulations. Error bars are SEM. (C) Mean average of relative Fas expression (from Figure 4B) *versus* mean average of relative 19-28ζ survival (from second stimulation readout in Figure S6B), r = Pearson correlation coefficient. (D) From experiment described in B, the percentage of surviving targets analysed after each target stimulation. Error bars are SEM, *P < 0.05, **P < 0.01, ****P < 0.0001, ns – non-significant, two-way ANOVA.

Figure S7

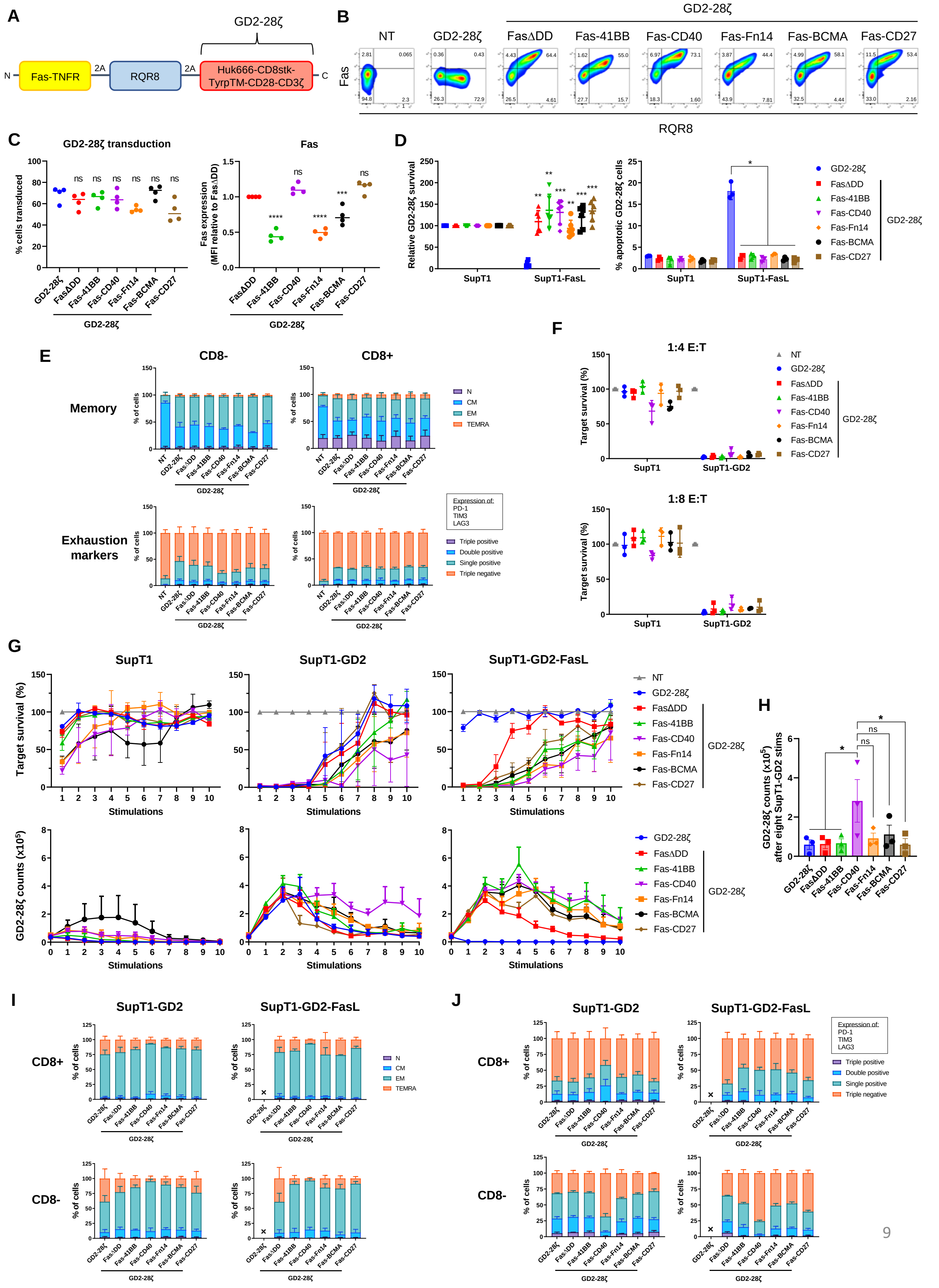
A
GD2-28ζ
N
Fas-TNFR
2A
RQR8
2A
Huk666-CD8stk-TyrpTM-CD28-CD3ζ
C
B
GD2-28ζ
NT
GD2-28ζ
FasΔDD
Fas-41BB
Fas-CD40
Fas-Fn14
Fas-BCMA
Fas-CD27
Fas
RQR8
C
GD2-28ζ transduction
% cells transduced
Fas
Fas expression (MFI relative to FasΔDD)
D
Relative GD2-28ζ survival
% apoptotic GD2-28ζ cells
SupT1
SupT1-FasL
E
CD8-
CD8+
Memory
Exhaustion markers
% of cells
N
CM
EM
TEMRA
Expression of: PD-1 TIM3 LAG3
Triple positive
Double positive
Single positive
Triple negative
F
1:4 E:T
1:8 E:T
Target survival (%)
SupT1
SupT1-GD2
NT
G
SupT1
SupT1-GD2
SupT1-GD2-FasL
Target survival (%)
GD2-28ζ counts (x10^5)
Stimulations
H
GD2-28ζ counts (x10^5) after eight SupT1-GD2 stims
I
J
CD8+
CD8-

Figure S7. Fas-TNFRs enhance GD2-28ζ CAR efficacy. (A) Schematic of polycistronic transgene transduced into human T cells. GD2-28ζ: Huk666 binder fused to the endodomains of CD28 and CD3ζ via a CD8 stalk and Tyrp transmembrane domain. (B) Representative flow cytometry plots from one human T-cell donor transduced to express either GD2-28ζ alone or co-express FasΔDD or the stated Fas-TNFRs. (C) Left: transduction percentages of T cells from four independent donors, ns – non-significant, one-way ANOVA (Dunnett's multiple comparisons test relative to GD2-28ζ). Right: MFI of the Fas-TNFRs relative to FasΔDD MFI, measured from top right quadrant in B. Four independent donors tested, mean being shown, ***$P < 0.001$, ****$P < 0.0001$, ns – non-significant, one-way ANOVA (Dunnett's multiple comparisons test relative to FasΔDD). (D) GD2-28ζ cells were cultured with SupT1^{FasKO} or SupT1^{FasKO}-FasL cells at a 1:1 E:T, either for 72 hours (left) or five hours (right), at which point GD2-28ζ cell survival or percentage of apoptotic cells (Annexin V^{+} 7AAD^{-}) were calculated, respectively. Six and three independent donors were tested for the cell survival and apoptotic analysis, respectively. Error bars are SEM, *$P < 0.05$, **$P < 0.01$, ***$P < 0.001$, two-way ANOVA. (E) Memory phenotype analysis (top) and percentage of exhaustion marker expression (bottom; PD-1, TIM3, LAG3) from GD2-28ζ cells co-expressing FasΔDD or the Fas-TNFRs under basal conditions. (F) GD2-28ζ cells co-cultured with SupT1 or SupT1-GD2 (Fas$^{+/+}$) target cells for 72 hours at 1:4 and 1:8 E:Ts, measuring for target survival. Three independent donors tested, error bars are SEM. (G) GD2-28ζ cells from three independent donors were stimulated up to ten times with either SupT1^{FasKO}, SupT1^{FasKO}-GD2 or SupT1^{FasKO}-GD2-FasL cells at a starting 1:1 E:T, measuring for target survival and GD2-28ζ cell counts after each stimulation. Effectors were stimulated with 50,000 targets for all ten stimulations, error bars are SEM. (H) GD2-28ζ cell counts after eighth round of SupT1^{FasKO}-GD2 stimulation, as described in G. *$P < 0.05$, ns – non-significant, two-way ANOVA, error bars are SEM. (I and J) After the tenth stimulation from experiment described in G, T-cell memory phenotype (I) and the percentage of GD2-28ζ cells expressing exhaustion markers: PD-1, TIM3 and LAG3 (J); were analysed. Error bars are SEM, an 'X' denotes where too few cells were present to accurately determine phenotype.

Figure S8

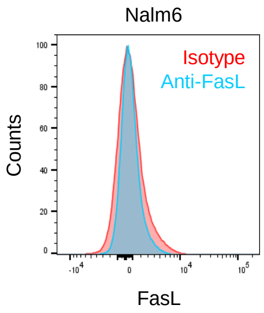

Figure S8. Nalm6 cells do not express FasL. Nalm6 cells were surface stained with an anti-FasL antibody or an isotype control antibody.

Table S1 – Electronic file showing NanoString® analysis comparing mRNA transcripts from cells expressing 19-ζ and FasΔDD or Fas-TNFRs *versus* cells expressing 19-ζ alone (baseline). Experiment performed as described in Figure 1F.

Table S2 – Electronic file showing NanoString® analysis comparing mRNA transcripts from cells expressing 19-ζ and Fas-TNFRs *versus* cells expressing 19-ζ and FasΔDD (baseline). Experiment performed as described in Figure 1F.
